# Subtyping-based platform guides precision medicine for heavily pretreated metastatic triple-negative breast cancer: The FUTURE phase II umbrella clinical trial

**DOI:** 10.1038/s41422-023-00795-2

**Published:** 2023-03-27

**Authors:** Yin Liu, Xiu-Zhi Zhu, Yi Xiao, Song-Yang Wu, Wen-Jia Zuo, Qiang Yu, A-Yong Cao, Jun-Jie Li, Ke-Da Yu, Guang-Yu Liu, Jiong Wu, Tao Sun, Jiu-Wei Cui, Zheng Lv, Hui-Ping Li, Xiao-Yu Zhu, Yi-Zhou Jiang, Zhong-Hua Wang, Zhi-Ming Shao

**Affiliations:** 1 Department of Breast Surgery, Fudan University Shanghai Cancer Center, Shanghai, China; 2 Key Laboratory of Breast Cancer in Shanghai, Department of Oncology, Shanghai Medical College, Fudan University, Shanghai, China; 3 Department of Medical Oncology, Cancer Hospital of China Medical University/Liaoning Cancer Hospital, Shenyang, Liaoning China; 4 Department of Medical Oncology, First Hospital of Jilin University, Changchun, Jilin, China; 5 Department of Breast Oncology, Key Laboratory of Carcinogenesis and Translational Research (Ministry of Education), Peking University Cancer Hospital & Institute, Beijing, China; 6 Jiangsu Hengrui Pharmaceuticals Co. Ltd, Shanghai, China

**Keywords:** Breast cancer, Tumour heterogeneity, Targeted therapies

## Abstract

Triple-negative breast cancer (TNBC) is a heterogeneous disease and lacks effective treatment. Our previous study classified TNBCs into four subtypes with putative therapeutic targets. Here, we report the final results of FUTURE, a phase II umbrella trial designed to explore whether the subtyping-based strategy may improve the outcomes in metastatic TNBC patients. A total of 141 patients with a median of three previous lines of therapies in the metastatic setting were enrolled in seven parallel arms. Confirmed objective responses were achieved in 42 patients (29.8%; 95% confidence interval [CI], 22.4–38.1). The median values of progression-free survival and overall survival were 3.4 (95% CI: 2.7–4.2) and 10.7 (95% CI: 9.1–12.3) months, respectively. Given Bayesian predictive probability, efficacy boundaries were achieved in four arms. Furthermore, integrated genomic and clinicopathological profiling illustrated associations of clinical and genomic parameters with treatment efficacy, and the efficacy of novel antibody–drug conjugates was explored in preclinical TNBC models of subtypes for which treatment was futile. In general, the FUTURE strategy recruits patients efficiently and provides promising efficacy with manageable toxicities, outlining a direction for further clinical exploration.

## Introduction

Triple-negative breast cancer (TNBC) is pathologically defined as a subgroup of breast cancers that lacks estrogen receptor (ER), progesterone receptor (PR) and human epidermal growth factor receptor 2 (HER2) expression;^1,2^ this precludes the use of targeted therapies, and the most available systemic treatment option is chemotherapy. Accounting for approximately 15% of invasive breast cancers, TNBC is associated with a high risk of early recurrence and poor patient outcome. The low response rate (5%–10%) of TNBC to standard chemotherapy in the later-line settings highlights the need for advances in therapeutic options.^3–5^

In recent years, immunotherapy has emerged as a breakthrough for treating TNBC,^6–8^ while PARP inhibitors have provided a significant benefit for patients carrying *BRCA* germline mutations.^9,10^ However, these treatments are far from satisfactory for metastatic TNBC due to the lack of evidence supporting immunotherapy in later-line treatment^11^ and the low prevalence of *BRCA* germline mutations.^12^ Recently, sacituzumab govitecan and fam-trastuzumab deruxtecan-nxki (DS-8201a; T-DXd; tradename Enhertu [Daiichi Sankyo]), two novel antibody–drug conjugates (ADCs), have been successively granted regular approval by the U.S. Food and Drug Administration for patients with metastatic TNBC and patients with metastatic HER2-low breast cancer, respectively.^4,13^ The identification of specific DNA alterations for available targeted therapies has also opened the door for genome-driven cancer treatment,^14–17^ but only a small fraction of TNBCs have targetable mutations.^18,19^ Owing to the overall poor prognosis and the complexity of molecular features of TNBC, there is an ongoing need to find effective therapeutic matches.

Studies describing targeted therapies for TNBC have laid the groundwork for precision medicine.^20–22^ However, those studies mainly focused on specific targets, ignoring the intrinsic subtypes of TNBCs and limiting the enrollment of TNBC patients without druggable targets, which reduced their clinical applicability. Our previous study presented multi-omic profiling of 465 Chinese TNBCs and classified them into four subtypes, namely, luminal androgen receptor (LAR), immunomodulatory (IM), basal-like immune-suppressed (BLIS) and mesenchymal-like (MES).^18^ Putative treatment options were then identified for each subtype, allowing for a broader population to achieve precision treatment. Building on that advancement, we developed an immunohistochemistry (IHC)-based classification approach, which simplified and increased the clinical utility of the subtyping system.^23^

Here, we aimed to assess the efficacy and safety of molecular subtyping and genomic sequencing-guided precision therapy for heavily pretreated metastatic TNBC. Our study recruited metastatic TNBC patients who were resistant to the most common chemotherapeutic agents used in breast cancer treatment.^1^ In the interim analysis, the outcomes were favorable, with an objective response rate (ORR) of 29.0% for 69 enrolled patients.^24^ Here, we report the final clinical efficacy (including survival data for the first time), safety profile, biomarker analysis, and exploration of optimized regimens of the Fudan University Shanghai Cancer Center TNBC umbrella (FUTURE) trial.

## Results

### Patient characteristics

**Fig. 1:**
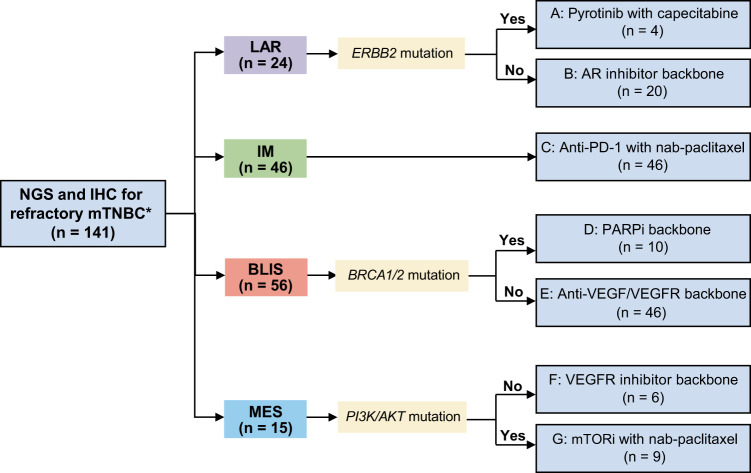
The FUTURE trial schema. *Patients with heavily pretreated mTNBC were stratified into seven arms using FUSCC NGS panel testing and IHC subtyping. NGS next-generation sequencing, IHC immunohistochemistry, mTNBC metastatic triple-negative breast cancer, FUSCC Fudan University Shanghai Cancer Center, LAR luminal androgen receptor, IM immunomodulatory, BLIS basal-like immune-suppressed, MES mesenchymal-like, AR androgen receptor, PD-1 programmed cell death-1, PARPi poly ADP-ribose polymerase inhibitor, VEGF vascular endothelial growth factor, VEGFR vascular endothelial growth factor receptor, mTORi mammalian target of rapamycin inhibitor.

Between October 18, 2018, and February 11, 2022, 141 patients were enrolled (Fig. 1). All patients were heavily pretreated (median of 3 previous lines of antitumor regimens in the metastatic setting [range, 1–8]), and most of them had received taxane (99.3%), anthracycline (92.9%), platinum (93.6%), vinorelbine (80.9%), capecitabine (87.9%) and gemcitabine (70.2%). The baseline characteristics are summarized in Table 1. The presence of germline *BRCA1/2* mutations and the expression of PD-L1 are shown in Supplementary information, Table S1, Table S2, respectively. The median age was 50 years (range, 23–74). Seventy-one (50.4%) patients had three or more metastatic sites, 69 (48.9%) patients had lung metastasis, and 43 (30.5%) patients had liver metastasis. At the data cutoff (March 31, 2022), the median follow-up time was 18.3 months (95% CI: 16.7–19.9). Nine patients continued to receive treatment in the trial. Detailed information and reasons for discontinuation are shown in Supplementary information, Fig. S1.

**Table 1:** Demographic and clinical characteristics at baseline.

Characteristics	ITT (*n* = 141)	A (*n* = 4)	B (*n* = 20)	C *(n* = 46)	D (*n* = 10)	E (*n* = 46)	F (*n* = 6)	G (*n* = 9)
Age at enrollment, median (range), year								
	50 (23–74)	59.5 (54–65)	54 (33–74)	50.5 (29–71)	46 (29–56)	49 (28–66)	47 (34–58)	51 (23–65)
ECOG performance status^a^, No. of patients (%)								
0	5 (3.5)	0	0	4 (8.7)	0	1 (2.2)	0	0
1	120 (85.1)	4 (100.0)	19 (95.0)	39 (84.8)	8 (80.0)	38 (82.6)	4 (66.7)	8 (88.9)
2	16 (11.3)	0	1 (5.0)	3 (6.5)	2 (20.0)	7 (15.2)	2 (33.3)	1 (11.1)
No. of previous therapies for metastatic disease, median (IQR)								
	3 (2–4)	4.5 (4–5)	3 (2–4)	3 (2–3)	3 (3–4)	3 (2–3)	2.5 (2–3)	3 (2–3)
Previous chemotherapy exposure, No. of patients (%)								
Taxane	140 (99.3)	4 (100.0)	20 (100.0)	46 (100.0)	9 (90.0)	46 (100.0)	6 (100.0)	9 (100.0)
Anthracycline	131 (92.9)	4 (100.0)	18 (90.0)	44 (95.7)	9 (90.0)	44 (95.7)	5 (83.3)	7 (77.8)
Platinum	132 (93.6)	4 (100.0)	19 (95.0)	40 (87.0)	10 (100.0)	44 (95.7)	6 (100.0)	9 (100.0)
Gemcitabine	99 (70.2)	3 (75.0)	16 (80.0)	30 (65.2)	6 (60.0)	34 (73.9)	5 (83.3)	5 (55.6)
Capecitabine	124 (87.9)	4 (100.0)	19 (95.0)	37 (80.4)	8 (80.0)	41 (89.1)	6 (100.0)	9 (100.0)
Vinorelbine	114 (80.9)	3 (75.0)	16 (80.0)	35 (76.1)	9 (90.0)	39 (84.8)	6 (100.0)	6 (66.7)
No. of metastases, No. of patients (%)								
1	21 (14.9)	0	7 (35.0)	5 (10.9)	1 (10.0)	5 (10.9)	2 (33.3)	1 (11.1)
2	49 (34.8)	2 (50.0)	4 (20.0)	19 (41.3)	2 (20.0)	16 (34.8)	2 (33.3)	4 (44.4)
≥ 3	71 (50.4)	2 (50.0)	9 (45.0)	22 (47.8)	7 (70.0)	25 (54.3)	2 (33.3)	4 (44.4)
Location of metastases, No. of patients (%)								
Lymph nodes	96 (68.1)	2 (50.0)	6 (30.0)	35 (76.1)	8 (80.0)	35 (76.1)	5 (83.3)	5 (55.6)
Lung	69 (48.9)	2 (50.0)	8 (40.0)	19 (41.3)	5 (50.0)	29 (63.0)	2 (33.3)	4 (44.4)
Liver	43 (30.5)	2 (50.0)	12 (60.0)	11 (23.9)	3 (30.0)	10 (21.7)	1 (16.7)	4 (44.4)
Bone	69 (48.9)	3 (75.0)	8 (40.0)	21 (45.7)	5 (50.0)	25 (54.3)	3 (50.0)	4 (44.4)
Chest wall	51 (36.2)	1 (25.0)	7 (35.0)	15 (32.6)	5 (50.0)	20 (43.5)	1 (16.7)	2 (22.2)
Breast	32 (22.7)	1 (25.0)	4 (20.0)	12 (26.1)	4 (40.0)	7 (15.2)	1 (16.7)	3 (33.3)
Others	30 (21.3)	0	5 (25.0)	11 (23.9)	4 (40.0)	8 (17.4)	2 (33.3)	0
Duration of first-line therapy, No. of patients (%)								
< 3 months	48 (34.0)	1 (25.0)	6 (30.0)	14 (30.4)	2 (20.0)	19 (41.3)	2 (33.3)	4 (44.4)
3–6 months	42 (29.8)	2 (50.0)	6 (30.0)	13 (28.3)	4 (40.0)	16 (34.8)	1 (16.7)	0
> 6 months	35 (24.8)	1 (25.0)	3 (15.0)	14 (30.4)	2 (20.0)	8 (17.4)	2 (33.3)	5 (55.6)
Unknown	16 (11.3)	0	5 (25.0)	5 (10.9)	2 (20.0)	3 (6.5)	1 (16.7)	0
Disease-free interval^b^, No. of patients (%)								
< 6 months	53 (37.6)	2 (50.0)	5 (25.0)	15 (32.6)	3 (30.0)	24 (52.2)	3 (50.0)	1 (11.1)
6–12 months	17 (12.1)	1 (25.0)	5 (25.0)	5 (10.9)	0	5 (10.9)	1 (16.7)	0
> 12 months	47 (33.3)	1 (25.0)	7 (35.0)	16 (34.8)	3 (30.0)	14 (30.4)	2 (33.3)	4 (44.4)
Initially diagnosed as stage IV	24 (17.0)	0	3 (15.0)	10 (21.7)	4 (40.0)	3 (6.5)	0	4 (44.4)

*ITT* Intention-to-treat, *ECOG* Eastern Cooperative Oncology Group, *IQR* interquartile range.

^a^Scores on the ECOG scale range from 0 (no disability) to 5 (death).

^b^Interval between the time of metastasis or relapse and the last dose of adjuvant chemotherapy or surgery (for neoadjuvant chemotherapy cases without adjuvant chemotherapy).

### Primary and secondary outcomes of the whole cohort

**Fig. 2:**
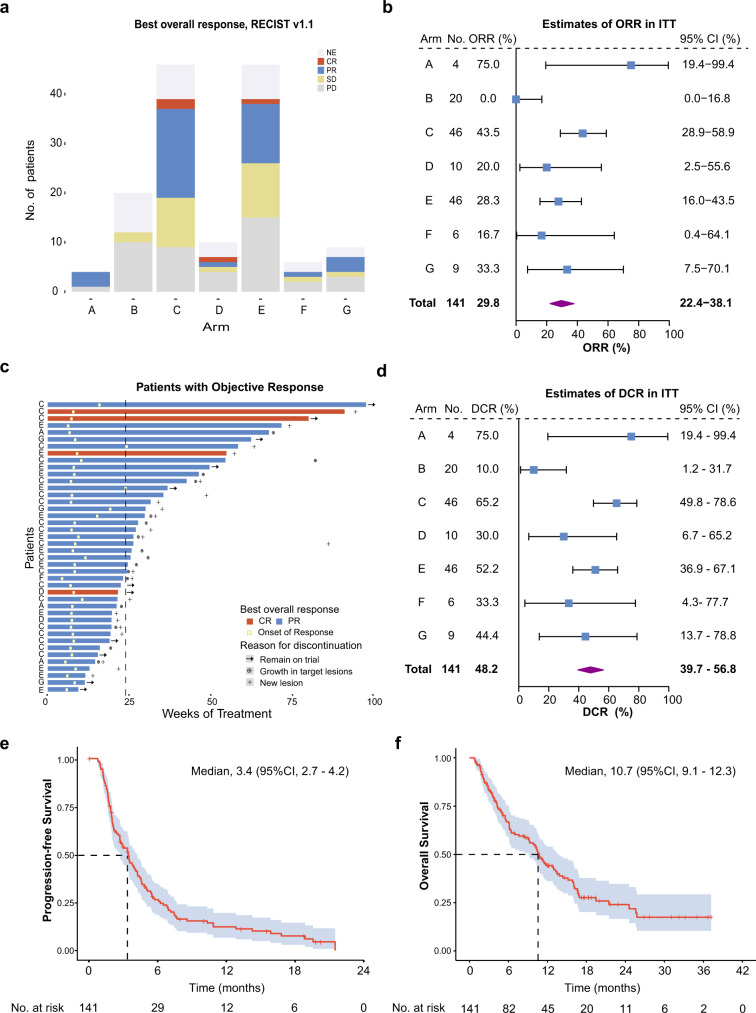
Summary of the primary and secondary endpoints. **a** Summary of confirmed responses according to RECIST v1.1 in each arm in the ITT population. **b** Forest plots of the ORR and 95% CI of each arm in the ITT population. **c** Durability of response among 42 patients with a confirmed objective response. The dashed line is at 24 weeks of treatment. **d** Forest plots of the DCR and 95% CI of each arm in the ITT population. **e**, **f** Kaplan‒Meier analysis of PFS (**e**) and OS (**f**) in the ITT population. RECIST response evaluation criteria in solid tumors, NE not evaluated, CR complete response, PR partial response, SD stable disease, PD progressive disease, ORR objective response rate, ITT intention-to-treat, CI confidence interval, DCR disease control rate.

Patients were enrolled into one of the following arms based on their TNBC subtypes and genomic features: (A) pyrotinib with capecitabine, (B) androgen receptor inhibitor backbone therapy, (C) anti-PD-1 with nab-paclitaxel, (D) PARP inhibitor backbone therapy, (E) anti-VEGF/VEGFR backbone therapy, (F) VEGFR inhibitor backbone therapy, and (G) mTOR inhibitor with nab-paclitaxel (Fig. 1). A total of 112 of the 141 enrolled patients underwent at least one postbaseline assessment (Supplementary information, Fig. S2), and the reasons for the remaining 29 patients not undergoing postbaseline assessments are listed in Supplementary information Table S3. In general, an objective response (complete response [CR] and partial response [PR]) was achieved in 42 (29.8%; 95% CI: 22.4–38.1) patients (Fig. 2a, b and Table 2), with a median time to response of 1.8 months and a median duration of response of 4.9 months. At the data cutoff, nine patients had long-term responses for more than 12 (range, 13.6–19.7) months (Fig. 2c). Disease control was achieved in 68 (48.2%; 95% CI: 39.7–56.8) patients (Fig. 2d).

**Table 2:** Tumor responses and survival outcomes.

Efficacy	ITT (*n* = 141)	A (*n* = 4)	B (*n* = 20)	C (*n* = 46)	D (*n* = 10)	E (*n* = 46)	F (*n* = 6)	G (*n* = 9)
CR^a^, No. of patients (%)	4 (2.8)	0	0	2 (4.3)	1 (10.0)	1 (2.2)	0	0
PR^a^, No. of patients (%)	38 (27.0)	3 (75.0)	0	18 (39.1)	1 (10.0)	12 (26.1)	1 (16.7)	3 (33.3)
SD, No. of patients (%)	26 (18.4)	0	2 (10.0)	10 (21.7)	1 (10.0)	11 (23.9)	1 (16.7)	1 (11.1)
PD, No. of patients (%)	44 (31.2)	1 (25.0)	10 (50.0)	9 (19.6)	4 (40.0)	15 (32.6)	2 (33.3)	3 (33.3)
NA^b^, No. of patients (%)	29 (20.6)	0	8 (40.0)	7 (14.3)	3 (30.0)	7 (15.2)	2 (33.3)	2 (22.2)
Objective response^c^								
No. of patients	42	3	0	20	2	13	1	3
Percentage of patients (95% CI)	29.8 (22.4–38.1)	75.0 (19.4–99.4)	0	43.5 (28.9–58.9)	20.0 (2.5–55.6)	28.3 (16.0–43.5)	16.7 (0.4–64.1)	33.3 (7.5–70.1)
Disease control^d^								
No. of patients	68	3	2	30	3	24	2	4
Percentage of patients (95% CI)	48.2 (39.7–56.8)	75.0 (19.4–99.4)	10.0 (1.2–31.7)	65.2 (49.8–78.6)	30.0 (6.7–65.3)	52.2 (36.9–67.1)	33.3 (4.3–77.7)	44.4 (13.7–78.8)
Median PFS (95% CI), month	3.4 (2.7–4.2)	3.4 (0–7.3)	1.9 (1.7–2.1)	4.6 (3.4–5.9)	2.0 (1.7–2.3)	3.4 (1.7–5.0)	1.2 (0–2.5)	3.0 (2.4–3.6)
Median OS (95% CI), month	10.7 (9.1–12.3)	16.7 (0–35.3)	6.1 (2.8–9.4)	16.1 (11.7–20.5)	6.2 (1.9–10.5)	10.1 (3.8–16.3)	2.7 (0–17.0)	4.5 (2.4–6.6)

*ITT* Intention-to-treat, *CR* complete response, *PR* partial response, *SD* stable disease, *PD* progressive disease, *NA* not available, *CI* confidence intervals, *PFS* progression free survival, *OS* overall survival.

^a^Confirmed responses only.

^b^Signifies patients who discontinued therapy before the first postbaseline scan because of progressive disease or a treatment-related adverse event.

^c^Defined as CR + PR.

^d^Defined as CR + PR + SD.

Moreover, we formally disclosed the survival data of the FUTURE trial. The median progression-free survival (PFS) was 3.4 (95% CI: 2.7–4.2) months; the estimated probability of PFS was 26.5% (95% CI: 22.4–30.6) at 6 months and 12.4% (95% CI: 9.2–15.6) at 12 months (Fig. 2e). At the data cutoff, 63.8% (90/141) of overall survival (OS) events were recorded. The median OS was 10.7 (95% CI: 9.1–12.3) months; the estimated probability of survival was 66.8% (95% CI: 62.7–70.9) at 6 months and 44.2% (95% CI: 39.7–48.7) at 12 months (Fig. 2f). Detailed survival events and censoring proportions of each arm are described in Supplementary information, Table S4.

### Arms achieving prespecified efficacy boundaries: A, C, E, G

**Fig. 3:**
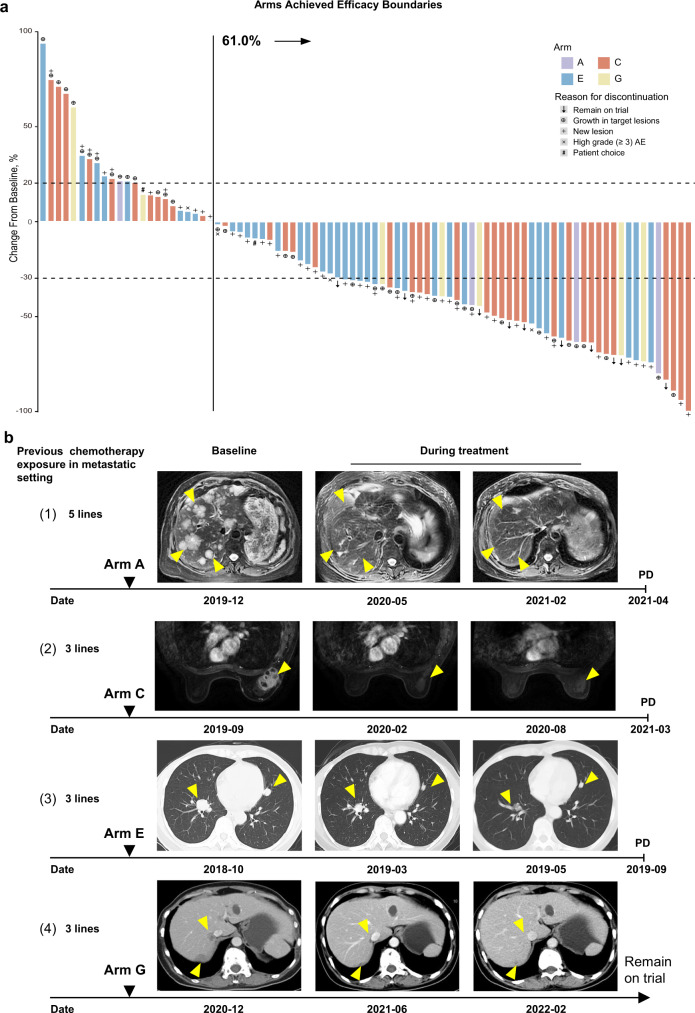
Tumor responses in arms that achieved prespecified efficacy boundary. **a** Best percent change in the sum of target lesion diameters (longest diameter for nonnodular lesions and short axis for nodal lesions) from baseline for 105 patients in arms A, C, E, G. The dashed lines at +20% and −30% indicate thresholds for progressive disease and partial response, respectively, according to RECIST v1.1. Eighty-seven patients in the 4 arms with postbaseline tumor assessments of target lesions are represented in the plot, while 18 patients without postbaseline tumor assessments of target lesions are not shown. **b** Examples of patients with objective responses. Yellow arrows indicate metastatic lesions. (1) A 66-year-old woman with mTNBC that progressed after 5 lines of previous treatment in the metastatic setting was identified as having the LAR subtype with an ERBB2 D769Y mutation; this patient was enrolled in arm A (December 2019). She received pyrotinib and capecitabine therapy and achieved a PR 1.6 months after the initiation of therapy. In April 2021, her intrahepatic lesions progressed. Baseline images of intrahepatic diffuse metastases and typical images of tumor regression during treatment are shown. (2) A 70-year-old woman with mTNBC that progressed after 3 lines of previous treatment in the metastatic setting was identified as having the IM subtype; this patient was enrolled in arm C (September 2019). She received anti-PD-1 therapy combined with nab-paclitaxel and achieved a PR 2.0 months after the initiation of therapy. She discontinued nab-paclitaxel and immunotherapy due to AEs in February and September 2020, respectively. She was followed up closely until March 2021, when her breast lesion had progressed. Images of the target lesion (right breast) before and during treatment are shown. (3) A 53-year-old female with mTNBC that progressed after 3 lines of previous therapy in the metastatic setting was identified as having the BLIS subtype; this patient was enrolled in arm E (October 2018). She received apatinib monotherapy and achieved a PR 1.9 months after the initiation of therapy. In September 2019, her lung metastases progressed. Computed tomography scans before and during treatment are shown. (4) A 54-year-old woman with mTNBC that progressed after 3 lines of previous treatment in the metastatic setting was identified as having the MES subtype with a PIK3CA H1047R mutation; this patient was enrolled in arm G (January 2021). She received everolimus with nab-paclitaxel therapy and achieved a PR 2.0 months after the initiation of therapy. She discontinued nab-paclitaxel chemotherapy after 10 cycles of therapy due to grade II neurotoxicity. In close follow-up, the patient’s disease remained stable for 14 months until March 2022. Images of the patient’s liver metastases before and during treatment are shown. AE adverse event, PD progressive disease, mTNBC metastatic triple-negative breast cancer, LAR luminal androgen receptor, IM immunomodulatory, BLIS basal-like immune-suppressed, MES mesenchymal-like.

According to the study design, arms A, C, E, and G reached the efficacy boundaries based on Bayesian prediction probability (see the “Bayesian prediction probability” part of the Materials and Methods section; more details in Supplementary information, Data S1).^25^ As shown in the waterfall plot, which depicted tumor responses in patients of arms A, C, E, and G with at least one response assessment, a reduction in target lesions was achieved in 61.0% (64/105) of patients (Fig. 3a). Examples of patients achieving objective responses are shown in Fig. 3b.

Four patients who had the LAR subtype with *ERBB2* mutation identified by next-generation sequencing (NGS) were enrolled in arm A, and confirmed objective responses were achieved in 3 patients, with a median PFS of 3.4 (95% CI: 0–7.3) months and a median OS of 16.7 (95% CI: 0–35.3) months. Arm A was terminated early due to its high response rate and the low prevalence of *ERBB2* alteration.

Forty-six patients who had the IM subtype were enrolled in arm C and received camrelizumab plus nab-paclitaxel. Arm C was also terminated early as it reached the prespecified sample size (*n* = 46, more details in Supplementary information, Data S1) and tumor responses are shown in Supplementary information, Fig. S3a. In general, arm C achieved an ORR of 43.5% (95% CI: 28.9–58.9; Fig. 2a, b), with a median PFS of 4.6 (95% CI: 3.4–5.9) months and a median OS of 16.1 (95% CI: 11.7–20.5) months (Supplementary information, Table S4). Of the 20 patients meeting confirmed objective responses, the median duration of response was 8.6 (range 1.2–19.7) months (Supplementary information, Fig. S3b, c).

Forty-six patients who had the BLIS subtype without germline *BRCA1/2* mutation were enrolled in arm E to receive anti-VEGF/VEGFR backbone therapy. Thirteen patients achieved confirmed objective responses, with 1 CR and 12 PRs (Supplementary information, Fig. S3d). The ORR was 28.3% (95% CI: 16.0–43.5; Fig. 2a, b), with a median PFS of 3.4 (95% CI: 1.7–5.0) months and a median OS of 10.1 (95% CI: 3.8–16.3) months (Supplementary information, Table S4). Among the 13 patients meeting confirmed objective responses, the median duration of response was 4.2 (range 0.9–15.3) months (Supplementary information, Fig. S3e, f).

Nine patients who had the MES subtype with *PI3K/AKT* mutation were enrolled in arm G, and confirmed objective responses were achieved in 3 patients, with a median PFS of 3.0 (95% CI: 2.4–3.6) months and a median OS of 4.5 (95% CI: 2.4–6.6) months (Supplementary information, Table S4).

### Arms not achieving prespecified efficacy boundaries: B, D, F

According to the study design, arms B, D, and F did not reach the efficacy boundaries (see the “Bayesian prediction probability” part of the Materials and Methods section; more details in Supplementary information, Data S1). Twenty patients who had the LAR subtype without *ERBB2* mutations were enrolled in arm B and received an AR inhibitor as the backbone of their treatment. Despite multiple adjustments to the combination regimen of AR inhibitors (more details in Supplementary information, Data S1), none of the patients achieved confirmed objective responses.

Arms D and F remained unclosed according to the study protocol until data cutoff. Ten patients who had the BLIS subtype with *BRCA* germline mutations were enrolled in arm D, and confirmed objective responses were achieved in two patients. Six patients who had the MES subtype without *PI3K/AKT* mutations were enrolled in arm F, and a confirmed objective response was achieved in only one patient. Detailed survival data for arms D and F are shown in Table 2 and Supplementary information, Table S4.

### Safety

Safety data were consistent with the known safety profiles of relevant drugs.^24,26^ No grade 5 treatment-related adverse events (TRAEs) were reported. The most common grade 3–4 adverse events (AEs) were leukopenia (15.6%), neutropenia (14.9%), anemia (12.1%), and thrombocytopenia (9.9%), while the most common grade 3–4 nonhematologic events were hypertension (5.0%) and proteinuria (2.8%). AEs of any grade occurring in more than 10% of patients and grade 3–4 AEs occurring in ≥ 1 patient are summarized in Table 3. The treatment discontinuation (due to AE) rate was 8.5%, and the dose reduction and/or delay rate was 23.4%. The number of AEs of any grade and grade 3–4 occurring in each arm are shown in Supplementary information, Table S5.

**Table 3:** Summary of treatment-related AEs.

AE^a^	ITT (*n* = 141)		A (*n* = 4)		B (*n* = 20)		C (*n* = 46)		D (*n* = 10)		E (*n* = 46)		F (*n* = 6)		G (*n* = 9)	
	Any	Grade ≥ 3	Any	Grade ≥ 3	Any	Grade ≥ 3	Any	Grade ≥ 3	Any	Grade ≥ 3	Any	Grade ≥ 3	Any	Grade ≥ 3	Any	Grade ≥ 3
	*n* (%)	*n* (%)	*n* (%)	*n* (%)	*n* (%)	*n* (%)	*n* (%)	*n* (%)	*n* (%)	*n* (%)	*n* (%)	*n* (%)	*n* (%)	*n* (%)	*n* (%)	*n* (%)
Nausea	33 (23.4)	0	1 (25.0)	0	2 (10.0)	0	6 (13.0)	0	5 (50.0)	0	18 (39.1)	0	1 (16.7)	0	0	0
Anorexia	21 (14.9)	3 (2.1)	2 (50.0)	0	0	0	2 (4.3)	1 (2.2)	1 (10.0)	0	12 (26.1)	2 (4.3)	4 (66.7)	0	0	0
Vomiting	20 (14.2)	0	1 (25.0)	0	2 (10.0)	0	4 (8.7)	0	3 (30.0)	0	10 (21.7)	0	0	0	0	0
Diarrhea	14 (9.9)	1 (0.7)	3 (75.0)	0	2 (10.0)	1 (5.0)	3 (6.5)	0	0	0	6 (13.0)	0	0	0	0	0
Constipation	3 (2.1)	0	0	0	0	0	1 (2.2)	0	1 (10.0)	0	1 (2.2)	0	0	0	0	0
Dyspnea	6 (4.3)	1 (0.7)	0	0	1 (5.0)	1 (5.0)	1 (2.2)	0	0	0	3 (6.5)	1 (2.2)	1 (16.7)	0	0	0
Cough	9 (6.4)	0	0	0	0	0	2 (4.3)	0	0	0	5 (10.9)	0	2 (33.3)	0	0	0
Fatigue	40 (28.4)	0	1 (25.0)	0	3 (15.0)	0	12 (26.1)	0	1 (10.0)	0	19 (41.3)	0	2 (33.3)	0	2 (22.2)	0
Pyrexia	8 (5.7)	2 (1.4)	0	0	0	0	5 (10.9)	1 (2.2)	0	0	1 (2.2)	0	1 (16.7)	0	1 (11.1)	1 (11.1)
Weight loss	13 (9.2)	0	2 (50.0)	0	0	0	2 (4.3)	0	0	0	8 (17.4)	0	0	0	1 (11.1)	0
Rash	10 (7.1)	1 (0.7)	0	0	0	0	6 (13.0)	1 (2.2)^b^	0	0	4 (8.7)	0	0	0	0	0
Arthralgia	6 (4.3)	0	0	0	1 (5.0)	0	2 (4.3)	0	1 (10.0)	0	2 (4.3)	0	0	0	0	0
Myalgia	4 (2.8)	0	0	0	0	0	3 (6.5)	0	1 (10.0)	0	0	0	0	0	0	0
Back pain	3 (2.1)	1 (0.7)	0	0	1 (5.0)	0	0	0	0	0	1 (2.2)	0	1 (16.7)	1 (16.7)	0	0
Mucositis	20 (14.2)	0	2 (50.0)	0	2 (10.0)	0	3 (6.5)	0	1 (10.0)	0	6 (13.0)	0	3 (50.0)	0	3 (33.3)	0
PPE	20 (14.2)	3 (2.1)	1 (25.0)	0	0	0	2 (4.3)	0	0	0	17 (37.0)	4 (8.7)	1 (16.7)	0	0	0
Peripheral neuropathy	34 (24.1)	0	1 (25.0)	0	3 (15.0)	0	20 (43.5)	0	0	0	9 (19.6)	0	0	0	1 (11.1)	0
Pneumonia	8 (5.7)	2 (1.4)	0	0	1 (5.0)	0	2 (4.3)	0	0	0	1 (2.2)	0	0	0	4 (44.4)	2 (22.2)
Hypertension	29 (20.6)	7 (5.0)	0	0	0	0	2 (4.3)	0	1 (10.0)	0	22 (47.8)	6 (13.0)	3 (50.0)	1 (16.7)	1 (11.1)	0
Anemia	78 (55.3)	17 (12.1)	1 (25.0)	0	8 (40.0)	4 (20.0)	29 (63.0)	5 (10.9)	5 (50.0)	1 (10.0)	25 (54.3)	3 (6.5)	4 (66.7)	1 (16.7)	5 (55.6)	3 (33.3)
Leukopenia	82 (58.2)	22 (15.6)	1 (25.0)	0	6 (30.0)	0	34 (74.0)	12 (26.1)	4 (40.0)	1 (10.0)	25 (54.3)	6 (13.0)	4 (66.7)	1 (16.7)	8 (88.9)	2 (22.2)
Neutropenia	51 (36.2)	21 (14.9)	1 (25.0)	0	3 (15.0)	1 (5.0)	23 (50.0)	11 (23.9)	3 (30.0)	2 (20.0)	15 (32.6)	4 (8.7)	4 (66.7)	2 (33.3)	2 (22.2)	1 (11.1)
Thrombocytopenia	48 (34.0)	14 (9.9)	1 (25.0)	0	6 (30.0)	3 (15.0)	12 (26.1)	3 (6.5)	5 (50.0)	4 (40.0)	16 (34.8)	2 (4.3)	3 (50.0)	1 (16.7)	5 (55.6)	1 (11.1)
Elevated ALT/AST	35 (24.8)	3 (2.1)	1 (25.0)	0	5 (25.0)	0	10 (21.7)	2 (4.3)	0	0	13 (28.3)	2 (4.3)	3 (50.0)	0	3 (33.3)	0
Proteinuria	21 (14.9)	4 (2.8)	0	0	0	0	1 (2.2)	0	0	0	16 (34.8)	4 (8.7)	5 (83.3)	1 (16.7)	0	0
Urinary occult blood	2 (1.4)	1 (0.7)	0	0	0	0	0	0	1 (10.0)	1 (10.0)	0	0	1 (16.7)	0	0	0
RCCEP	28 (19.9)	1 (0.7)	0	0	0	0	28 (60.9)	1 (2.2)	0	0	0	0	0	0	0	0
Hypothyroidism	6 (4.3)	1 (0.7)	0	0	0	0	4 (8.7)	1 (2.2)	0	0	2 (4.3)	0	0	0	0	0
Hyperglycemia	3 (2.1)	1 (0.7)	0	0	0	0	2 (4.3)	1 (2.2)	0	0	1 (2.2)	0	0	0	0	0
Hypokalemia	1 (0.7)	1 (0.7)	1 (25.0)	1 (25.0)	0	0	0	0	0	0	0	0	0	0	0	0
Stroke	1 (0.7)	1 (0.7)	0	0	0	0	0	0	0	0	1 (2.2)	1 (2.2)	0	0	0	0
Edema limbs	9 (6.4)	0	0	0	0	0	7 (15.2)	0	0	0	2 (4.3)	0	0	0	0	0
Skin ulceration	3 (2.1)	3 (2.1)	0	0	0	0	0	0	0	0	3 (6.5)	3 (6.5)	0	0	0	0

*ITT* Intention-to-treat, *ALT* alanine aminotransferase, *AST* aspartate transaminase, *PPE* palmar-plantar erythrodysesthesia, *RCCEP* reactive cutaneous capillary endothelial proliferation.

^a^Events of any grade occurring in ≥ 10% of patients or grade 3–4 events occurring ≥ 1 patient are shown. No grade 5 events occurred.

^b^Bullous dermatitis.

### Post hoc biomarker analysis

**Fig. 4:**
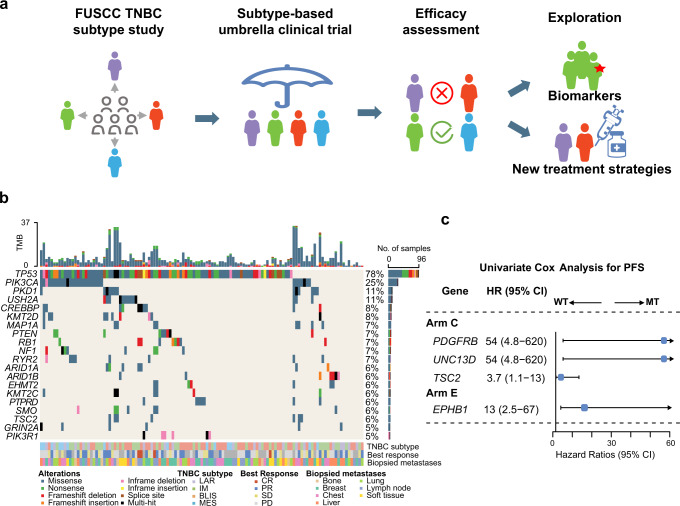
Potential predictive biomarkers of treatment efficacy. **a** A sketch map showing the subtype-based comprehensive study of FUSCC TNBC precision medicine. **b** Genomic landscape of refractory TNBC patients. **c** An exploratory forest plot of PFS in each arm and genes significantly affecting PFS are shown. Somatic mutations significantly affecting PFS were found only in arms C and E. Specific somatic mutations and unstratified hazard ratios with 95% CIs for PFS were obtained by univariate Cox hazard analysis. FUSCC Fudan University Shanghai Cancer Center, TNBC triple-negative breast cancer, LAR luminal androgen receptor, IM immunomodulatory, BLIS basal-like immunesuppressed, MES mesenchymal-like, CR complete response, PR partial response, SD stable disease, PD progressive disease, PFS progressionfree survival, HR hazard ratios, CI confidence interval, WT wild-type, MT mutation.

We then explored clinical features and genomic events associated with treatment response and tested the efficacy of novel ADCs in different subtypes of TNBCs to further inform precision oncology (Fig. 4a).

We first evaluated the ORR in a variety of clinical subgroups in the FUTURE trial. Patients with more than 3 different organ metastases had a significantly lower ORR (17.1% [95% CI: 7.2–32.1] vs 35.0% [95% CI: 25.7–45.2]), while those over 50 years of age at diagnosis had a higher ORR (39.4% [95% CI: 28.0–51.7] vs 20.0% [95% CI: 11.4–31.3]) (Supplementary information, Table S6). Genomic analysis included 129 (91.5%) patients with Fudan University Shanghai Cancer Center (FUSCC) NGS sequencing. The most prevalent somatic variations were *TP53* (78%), *PIK3CA* (25%) and *PKD1* (11%) (Fig. 4b). High mutation frequencies were observed in the genome integrity (83%), PI3K signaling (44%) and RTK signaling (25%) pathways (Supplementary information, Fig. S4). We then used univariate Cox regression analyses to explore the predictive value of frequent somatic mutations (≥ 5%) for PFS in each arm. Interestingly, a reduced clinical benefit of immunotherapy (arm C) was observed in patients with *PDGFRB*, *UNC13D* or *TSC2* mutations; patients with *EPHB1* mutation had shorter PFS in arm E (Fig. 4c). Additionally, we analyzed the genomic characteristics of patients treated with everolimus for *PIK3CA* mutation (B and G arms). Despite the similar *PI3KCA* mutation site (seven with H1047R and one with H1047L), the favorable outcome of everolimus was observed only in arm G (Supplementary information, Fig. S5), further suggesting the need for a subtyping-based precision treatment strategy.

### ADCs provide great efficacy in BLIS and LAR subtypes

**Fig. 5:**
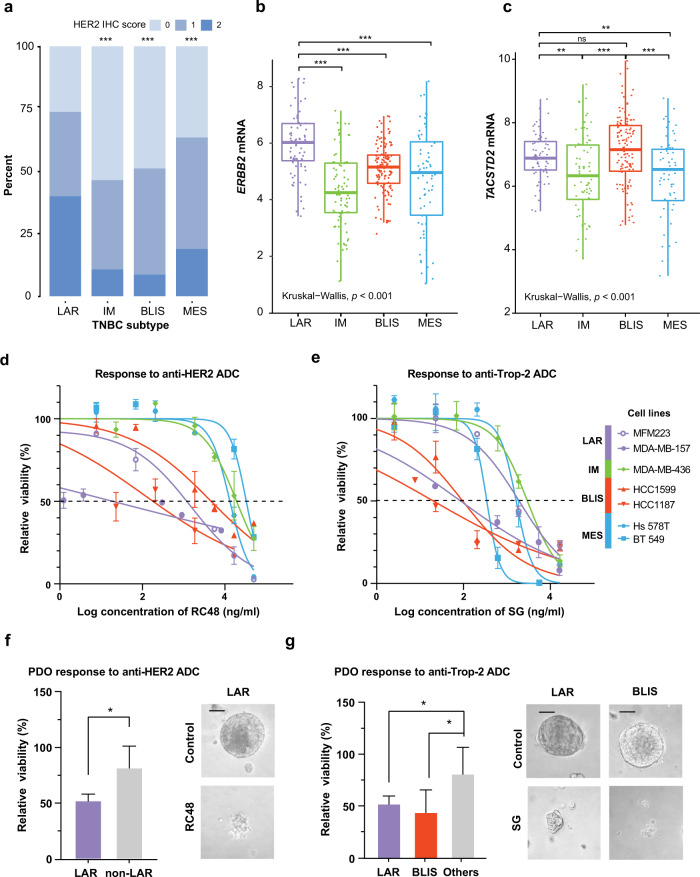
Potent antitumor activity of ADCs in LAR and BLIS subtypes. **a** IHC score of HER2 in TNBC molecular subtypes. *P* values were calculated by the chi-square test. **b**, **c** *ERBB2* (**b**) and *TACSTD2* (**c**) mRNA expression in TNBC molecular subtypes. *P* values were calculated by Wilcoxon’s test and Kruskal–Wallis test. **d**, **e** Growth curves of different subtypes of TNBC cell lines in response to the anti-HER2 ADC RC48 (**d**) and the anti-Trop-2 ADC sacituzumab govitecan (**e**). **f**, **g** Viability of PDOs in response to the anti-HER2 ADC RC48 at 0.5 μg/mL (**f**) and the anti-Trop-2 ADC sacituzumab govitecan at 1.0 μg/mL (**g**). *P* values were calculated by two-tailed Student’s *t*-test. Scale bars, 100 μm. IHC immunohistochemistry, LAR luminal androgen receptor, IM immunomodulatory, BLIS basal-like immune-suppressed, MES mesenchymallike, ADC antibody‒drug conjugate, SG sacituzumab govitecan, PDO patient-derived organoid.

Considering the poor outcomes in BLIS and LAR subtype patients, we then tried to explore new treatment strategies for these patients. ADCs have shown strong antitumor activity in solid tumors, especially breast cancer.^27^ For breast cancer, anti-HER2 ADCs and anti-Trop-2 ADCs are promising.^4,13^ In our multi-omic data from a TNBC patient cohort (*n* = 360),^18^ we observed that the expression of *ERBB2* (encoding HER2 protein) was higher in the LAR subtype than in other subtypes, both at the protein (*P* < 0.001, Fig. 5a) and mRNA levels (*P* < 0.001, Fig. 5b), suggesting that patients diagnosed with the TNBC LAR subtype might benefit from anti-HER2 ADCs. Interestingly, in LAR and BLIS subtype patients, the mRNA expression of *TACSTD2* (encoding Trop-2 protein) was higher than that in IM and MES subtype patients, suggesting that the LAR and BLIS subtypes might be sensitive to anti-Trop-2 ADC (*P* < 0.001, Fig. 5c). Subsequently, we investigated the efficacy of these two ADCs in TNBC cell lines and patient-derived organoids (PDOs) grouped by subtype (Fig. 5d–g; Supplementary information, Fig. S6a). Notably, both models showed that the LAR subtype had lower IC_50_ and viability in response to anti-HER2 ADC RC48 (Fig. 5d, f). In addition, tumors of LAR and BLIS subtypes had better responses to anti-Trop-2 ADC sacituzumab govitecan (SG) (Fig. 5e, g). Moreover, the expression levels of HER2 and Trop-2 in the TNBC cell lines and PDOs correlated with the ADCs efficacy (Supplementary information, Figs. S6b, S7). Collectively, ADCs showed great efficacy in BLIS and LAR subtypes, holding promise for future design of precision strategies.

## Discussion

In this prospective umbrella trial, we evaluated the feasibility and clinical utility of the subtyping-based precision strategy in heavily pretreated metastatic TNBC patients. Our work established a subtyping platform to navigate the precision treatment of TNBC based on the recognition of molecular characteristics instead of genomic alterations. The main purpose of this work was to highlight the superiority of the platform, not the superiority of a specific drug or regimen, as future advances in drug development could supplant the leading drugs. Notable accomplishments in this study include the following: (1) we demonstrated that TNBC subtyping combined with NGS was clinically feasible for matching and enrolling patients, with new biomarker-driven treatment arms being introduced and conducted simultaneously after previous arms reached a futility or efficacy boundary; (2) promising outcomes were confirmed in a subtype and genomic characteristics dual-directed therapeutic strategy, and these outcomes can be translated into long-term survival benefit; (3) integrated genomic and clinicopathological profiling illustrated associations of clinical and genomic parameters with treatment efficacy, and for arms with unsatisfactory response, novel ADCs were tested, providing clues for further exploration.

The FUTURE study recruited 141 TNBC patients. Unlike most umbrella trials focusing on specific therapies,^14,15,28^ this study was mainly driven by molecular subtyping rather than single gene alterations. Therefore, because of treatment allocation according to molecular subtype, FUTURE allowed enrollment of more screened patients (93.4% [141/151] compared with a 10%–20% enrollment rate in most biomarker-driven studies), which was critical for mTNBC patients with limited treatment options after progression on multiple lines of chemotherapy. In addition, Bayesian predictive probability was adopted to make adequacy of sample size of each arm more flexible,^25^ allowing the potential efficacy of the drug combination to be tested quickly and efficiently, especially in the arms with relatively low enrollment rates.

In addition, patients enrolled in this study were heavily pretreated and chemotherapy resistant with a short disease-free interval and duration of first-line therapy. In this study, the ORR reached nearly 30%, the median PFS reached 3.4 months, and the median OS reached 10.7 months, all of which were more favorable than the outcomes of traditional chemotherapy in the heavily pretreated patients of the TNBC population (ORR of 5%, median PFS of 1.7 months, and median OS of 6.7 months).^4^ Among the reported 7 arms, arms A, C, E, and G reached an efficacy boundary and arm B reached a futility boundary, while the data from arms D and F were immature due to the low proportion of these 2 specific subgroups. The imbalance of patient numbers between different arms may reflect the natural distribution of TNBC molecular subtypes in the metastatic setting as arms D and F remained open during the whole recruitment.

Arms A, C, E, and G demonstrated promising outcomes. For arm C, the results for PD-1 blockade plus chemotherapy in the IM subtype showed the highest ORR reported in a prospective trial conducted with heavily pretreated metastatic TNBC patients. A highlight of arm C was the usage of CD8 to define the IM subtype and match “immune-hot” tumors, validating the initial hypothesis that we proposed.^18^ Arm C achieved an ORR of 43.5%, with a median PFS of 4.6 months and a median OS of 16.1 months. In addition, the favorable response was remarkably durable, with a median duration of 8.6 months. This was also validated in the FUTURE-C-Plus trial, where the camrelizumab backbone regimen achieved a confirmed ORR of 81.3% and a median PFS of 13.6 months in the first-line treatment of CD8^+^ advanced TNBC patients.^29,30^ The subsequent randomized controlled phase III study (NCT05134194) is ongoing.

BLIS is characterized by high expression of the VEGF signature, which is associated with tumor angiogenesis and poor prognosis.^18^ In patients with the BLIS subtype without *BRCA* germline mutation in arm E, we assessed the effect of anti-VEGF/VEGFR therapy. A confirmed ORR of nearly 30% was achieved, which was higher than previously reported results in heavily pretreated TNBC patients.^31^ These findings suggested that anti-VEGF/VEGFR therapy showed preliminary efficacy in *BRCA* wild-type BLIS tumors, and it warrants further exploitation in *BRCA*-mutated patients. Bevacizumab or low-dose apatinib combined with VP-16 may be more tolerated than apatinib 500 mg.

Interestingly, arms A and G showed promising outcomes in a small sample size. In rare instances (2%–4%), patients with metastatic breast cancer have *ERBB2* mutations but are HER2-negative according to clinical guidelines.^32^ In patients with LAR subtypes, *ERBB2* mutations were enriched.^18^ Arm A achieved a confirmed ORR of 75% after receiving capecitabine plus pyrotinib. This arm suggested the potential of anti-HER2 therapy in tumors harboring *HER2* mutations. Similarly, the SUMMIT study demonstrated that neratinib combined with trastuzumab showed good antitumor activity in patients with *ERBB2*-mutated TNBC after previous multiline therapy, with an ORR of 33.3% and a median PFS of 6.2 months.^33^ A total of 9 MES patients with *PI3K/AKT* mutations were enrolled in arm G, and 3 of them achieved confirmed PRs. Similarly, the PAKT trial and the LOTUS trial showed that the addition of the AKT inhibitor capivasertib or ipatasertib to first-line paclitaxel therapy resulted in significantly longer PFS, with more pronounced benefits in *PIK3CA/AKT1/PTEN*-altered tumors.^34,35^ Conversely, IPATunity130 failed to repeat the benefit in HER2-negative patients.^36^ Overall, these two arms proved the utility of FUTURE to evaluate drug activity in patients harboring rare genomic aberrations in the context of molecular subtyping.

By comparison, treatment efficacy was unsatisfactory in arms B, D, and F. For BLIS with *BRCA* germline mutation, arm D tested the efficacy of PARP inhibitors plus famitinib, but only 2 patients responded, which might be attributable to prior usage of platinum. All patients in arm D had previously undergone treatment with platinum agents, and 8 of them had progressed during platinum treatment. Such patients were deemed resistant to PARP inhibitors and were excluded from clinical trials evaluating the efficacy of PARP inhibitors.^9,10^ Moreover, we experimentally demonstrated that anti-Trop-2 ADC had a strong inhibitory effect in the BLIS subtype, probably due to higher *TACSTD2* expression in BLIS. Hence, anti-Trop-2 ADC might be promising in the treatment of BLIS subtype patients.^37,38^ Meanwhile, although previous studies suggested that the LAR subtype was enriched with Chr9p21 loss, *CDKN2A* losses/deletions^18^ and somatic mutations in the PI3K signaling pathway,^39^ the overall efficacy of arm B was disappointing, with no patient responding to AR inhibitors with CDK4/6 inhibitors or mTOR inhibitors and with a median PFS of 1.9 months. In a recent study, bicalutamide plus abiraterone achieved a 19% clinical benefit rate in AR-positive advanced TNBC.^40,41^ This inconsistency may be due to tumor evolution and patient selection after multiline chemotherapy in our study; another possibility is that AR may only be a biomarker rather than a therapeutic target. Interestingly, we observed that the LAR subtype exhibited relatively higher *ERBB2* and *TACSTD2* expression. In vitro experiments confirmed that anti-HER2 and anti-Trop-2 ADCs had strong inhibitory effects in cell lines and organoids of LAR subtype patients. Therefore, these drugs may be promising in treating LAR subtype patients and should be tested in further studies. Finally, only six patients who had the MES subtype and no *PI3K/AKT* mutations were enrolled in arm F, which does not provide sufficient grounds for a conclusion at the current stage.

FUTURE is a pilot study designed to generate clues for clinical practice. Owing to the design of this umbrella trial, the number of patients is relatively small in some arms and unbalanced between arms, which has been discussed above. Notwithstanding the relatively limited sample size, this study offers valuable insights into tailoring and bolstering precision therapy through prospective molecular subtyping selection. Next, direct comparison with other chemotherapy approaches was not available in this trial because of its noncomparative design and the lack of accessible treatment options for the heavily pretreated patients of this population. Our randomized controlled umbrella FUTURE-SUPER trial (NCT04395989) is currently in progress. Finally, although PFS was not as satisfactory as sacituzumab govitecan, the primary endpoints ORR and OS were both comparable.^3^ Likewise, as the first step in tailoring treatment contingent on molecular subtyping in metastatic TNBC, the “FUTURE” strategy may constitute a platform to test the possibility of combining novel drugs within the framework of subtyping hereafter.

Collectively, the subtyping-based and genomic sequencing-guided strategy promotes promising efficacy with manageable toxicity in patients with heavily pretreated metastatic TNBC. As a dynamic and ongoing platform for novel targeted regimens, FUTURE can enable efficient testing of potential new drug–biomarker combinations in the context of subtyping, generating clues for further validation in expansion trials.

## Materials and methods

### Study design and participants

FUTURE is a phase II, open-label, multicenter, umbrella trial evaluating the efficacy and safety of multiple precision treatments based on molecular subtype and tumor characteristics in patients with heavily pretreated metastatic TNBCs. Eligibility criteria included the following: (1) female patients diagnosed with metastatic breast carcinoma with an ER-negative, PR-negative, and HER2-negative phenotype (the IHC cutoff for ER/PR-negative status was less than 1% staining in nuclei, and HER2-negative status was defined as a score of 0 or 1 by IHC analysis or the absence of ERBB2 amplification by fluorescence in situ hybridization with an IHC score); (2) central pathologic examination of tumor specimens performed by the Department of Pathology at FUSCC; (3) an Eastern Cooperative Oncology Group (ECOG) performance status of 0 to 2; (4) at least one measurable lesion according to Response Evaluation Criteria In Solid Tumors (RECIST) version 1.1; and (5) adequate hematologic function, hepatic function, and renal function. Patients with uncontrolled brain metastasis were excluded from enrollment. Full eligibility criteria are provided in the study protocol in Supplementary information, Data S1.

The trial was conducted in accordance with the Declaration of Helsinki and the Good Clinical Practice guidelines of the International Conference on Harmonization. The study protocol was approved by the institutional review board and ethics committee of FUSCC. The ethics committee reference number was 1807188-16. All patients provided written informed consent to participate in this study.

### Procedures

Patients who had heavily pretreated metastatic TNBCs and experienced disease progression during or following almost all standard chemotherapies (anthracycline, taxane, platinum, vinorelbine, capecitabine, and gemcitabine) were screened in four centers, including FUSCC, Beijing Cancer Hospital, Liaoning Cancer Hospital and Institute and the First Hospital of Jilin University. Tumor biopsies were obtained to allow IHC staining (AR, CD8 and FOXC1) and NGS (Supplementary information, Table S7)^23^ in order to classify the tumors into four subtypes (LAR, IM, BLIS, MES),^24^ and each patient was then enrolled into one of seven arms: (A) pyrotinib (HER1/HER2/HER4 inhibitor) 400 mg orally once daily continuously plus capecitabine 1000 mg/m^2^ orally twice daily from day 1 to day 14 on a 21-day cycle for LAR subtype with *ERBB2* somatic mutation or amplification; (B) AR inhibitor (SHR3680) 240 mg orally once daily backbone therapy for LAR subtype without *ERBB2* somatic mutation or amplification; (C) anti-PD-1 antibody (SHR1210) 200 mg intravenously once every 2 weeks with nab-paclitaxel 100 mg/m^2^ intravenously on days 1, 8, and 15 in a 28-day cycle for IM subtype; (D) PARP inhibitor (SH3162) 150 mg orally twice daily plus VEGFR inhibitor (famitinib) 20 mg orally once daily for BLIS subtype with *BRCA1/2* germline mutation; (E) anti-VEGF/VEGFR backbone therapy for BLIS subtype without *BRCA1/2* germline mutation; (F) VEGFR inhibitor (famitinib) orally once daily continuously plus etoposide (VP-16) 50 mg orally once daily from day 1 to day 14 in a 21-day cycle for MES subtype without *PI3K/AKT* mutation; (G) mTOR inhibitor (everolimus) 10 mg orally once daily continuously with nab-paclitaxel by intravenous 100 mg/m^2^ on days 1, 8, and 15 in a 28-day cycle for MES subtype with *PI3K/AKT* mutation. Details are provided in Table 5 of Supplementary information, Data S1.

### Bayesian predictive probability, sample size, and protocol modifications

Considering different degrees of enrollment efficiency and different population distributions among the treatment arms, we used the Bayesian predictive probability approach to lay out an adaptive design in the new protocol amendment (July 24, 2020). This study was originally designed to have 10–20 patients enrolled in each arm, bringing the estimated sample size to approximately 140 patients. Referring to the historical data of heavily pretreated TNBC patients after multiline chemotherapy,^42^ if three or more patients in each arm reached CR or PR, then the arm would be considered to have reached the efficacy boundary. Using Bayesian predictive probability, based on the number of patients who achieved objective response (CR + PR) in real time, each arm could be terminated independently according to futility or efficacy boundaries.^43^ Assuming that the reference objective response rate is p0 = 15%, the prior probability fits the beta distribution (0.05, 0.05). The final threshold value of 0.5 was adopted for the arm to achieve effectiveness, 0.1 was adopted as the threshold value for early termination due to ineffectiveness, and 0.9 was adopted as the threshold value for early termination due to effectiveness. Using Bayesian prediction probability, futility and efficacy boundaries were obtained, and the simulation results under different true values of ORR are shown in the Supplementary information, Data S1.

Due to the difficulty of enrollment and the promising efficacy observed in other arms in the interim analysis, arms A and G could be terminated early at fewer than 10 patients (more details in Supplementary information, Data S1). Arm C was expanded to a maximum of 41 cases based on the efficacy reported in the interim analysis. Considering a dropout rate of 10%, 46 patients needed to be enrolled.

Some modifications have been made in the new version of protocol, and we briefly summarized them as follows: (1) the sample sizes in each arm were set to be more flexible according to Bayesian prediction probability method, and the rationale behind it was explained as above, (2) CTCAE version 4.0 was updated to version 5.0, (3) bevacizumab or low dose VEGFR inhibitors have been explored due to the toxicity issues with the VEGFR inhibitors.

The study design allowed arms to be dynamic so that old arms could be eliminated when finished and new arms could be added. Notably, the investigators used FUTURE as a platform to test the safety and efficacy of potential new drug–biomarker combinations in heavily pretreated TNBC patients.

### Outcomes

The primary endpoint was the ORR per investigator according to RECIST v1.1 using imaging at baseline and every two cycles until disease progression.^44^ A CR or a PR was confirmed with one sequential tumor assessment at least 4 weeks later. Secondary endpoints were PFS (defined as the interval from the start of treatment to disease progression or death from any cause, whichever occurred first, or last PFS assessment for patients alive without progression), OS (first study dose until death from any cause), disease control rate (DCR, proportion of patients who experience a best response of CR or PR or stable disease ≥ 8 weeks according to RECIST version 1.1), and safety and tolerability. For PFS analysis, death before the first progressive disease (PD) assessment was computed as progressed, death between adequate assessment visits was computed as progressed, and death after more than one missed visit was censored on the date of last documented nonprogression. Treatment discontinuation for undocumented progression was censored on the date of last assessment without progression. Safety evaluations included assessments of AEs and serious AEs (SAEs), laboratory safety evaluations, vital signs, and physical examination. AEs were assessed in accordance with the National Cancer Institute Common Terminology Criteria for AEs, version 5.0. For AEs with various grades, the maximum reported grade was used in the summary table.

### Biospecimen collection, quality control, and processing

Tumor and matched blood DNA were isolated from tumor samples and peripheral lymphocytes using TGuide M24 (Tiangen, Beijing, China). Absorbance at 260 nm (A260) and 280 nm (A280) was measured to estimate the purity and quantity of the total DNA by a NanoDrop 2000 spectrophotometer (Thermo Scientific, Wilmington, DE, USA). The extracted DNA was considered suitable for subsequent experiments if the A260/A280 ratio was between 1.6 and 1.9.

### Sequencing using the FUSCC-BC panel

Details on the sequencing protocol have been described previously.^19^ The FUSCC breast cancer (FUSCC-BC) panel was used in this study (Supplementary information, Table S7). Both tumor and matched blood samples were sequenced. A KAPA HyperPlus kit (Kapa Biosystems) and Illumina HiSeq X TEN platform (Illumina Inc., San Diego, CA, USA) were used during NGS sequencing. Each alteration identified by the pipeline was manually reviewed to ensure that no false positives were reported.

### Genomic biomarker analysis

Somatic mutations were called from the tissue and blood BAM files using GATK4 Mutect2 with the default parameters. The VCF files were annotated using ANNOVAR. The variants and annotation results were transferred into Excel spreadsheets. Oncogenic signaling pathways were defined based on a previous study.^45^ In post hoc exploratory analyses, PFS for each cohort was analyzed by tissue somatic mutation status.^19^

### TNBC organoid and cell line classification

TNBC organoids were subjected to IHC staining (AR, CD8 and FOXC1) for subtyping.^23^ We performed hierarchical clustering to determine the TNBC subtype of common TNBC cell lines based on the similarity of expression profiles between patients and cell lines (Supplementary information, Fig. S6a). Patient RNA-seq data along with TNBC subtype annotations were obtained from our previous study.^18^ Cell line RNA-seq data were derived from the Cancer Cell Line Encyclopedia database and the study of Gong and colleagues.^46^ Cell lines with specific TNBC subtypes (according to the consistency of the FUSCC TNBC subtype and Lehmann subtype^20^) were chosen for further experiments.

### Cell proliferation assay

We used the human TNBC cell lines BT-549, HCC1187, HCC1599, Hs 578 T, MDA-MB-157, MDA-MB-436, and MFM223 (from ATCC). Cell proliferation assays were performed as previously described.^47,48^ Briefly, the cells of interest (1 × 10^3^–3 × 10^3^ cells per well) were seeded into 96-well plates overnight in 100 μL of complete growth medium and then treated with the indicated drugs for 5 days in triplicate. Cell viability was tested using the cell counting kit-8 (CCK-8) assay (Dojindo Molecular Technologies, Japan, CK04) according to the manufacturer’s instructions.

### Organoid preparation and culture

We developed a biobank for organoid storage as previously described.^46^ Briefly, fresh breast cancer tissues were minced into small fragments using sterile scalpels. Tissues were digested and resuspended in 10 mL of TAC buffer, incubated for 3 min to remove red blood cells and passed through a 100 mm cell strainer (Corning). For passaging, 5 mL of harvesting solution (Trevigen, 3700-100-01) was used to digest the basement membrane extract, which was incubated on ice for 1 h. Subsequently, the organoids were centrifuged at 350× *g* for 5 min, washed in digestion buffer and spun down. Next, 3 mL TrypLE Express (Invitrogen) was added, and organoids were incubated at room temperature for 3 min, followed by mechanical dissociation to small cell clusters by pipetting. Organoids were passaged at a 1:2–3 dilution every 2–3 weeks.

### Drug response test of TNBC organoids

Drug response testing of TNBC organoids was performed according to a previous paper.^49^ For organoid drug treatment, organoids in good condition were harvested and digested into single cells. Twenty-five microliters of organoid suspension was added to a cell-repellent black surface in clear bottom 384-well plates (Greiner 781976-SIN) with 1 × 10^3^–3 × 10^3^ cells per well and cultured for another 5–6 days before drug treatments. Organoids with ADCs were cultured for 2 weeks before testing for viability. Organoid cell viability was evaluated by a CellTiter-Glo 3D cell viability assay (Promega, G9683) according to the manufacturer’s instructions.

### PD-L1 and Trop-2 IHC

Baseline PD-L1 expression in the FUTURE trial was assessed at a central laboratory and characterized according to the combined positive score (CPS) as reported previously.^11^ CPS was defined as the ratio of PD-L1-positive cells (tumor cells, lymphocytes, and macrophages) out of the total number of tumor cells multiplied by 100. Available tumor specimens were stained for Trop-2 by IHC as reported previously.^37,50^ Positivity required at least 10% of the tumor cells to be stained.

### Statistical analysis

The primary efficacy analysis population was the intention-to-treat population, including all eligible patients enrolled in the study. Safety was analyzed in all patients who received at least one dose of the study medication. The ORR and DCR with 95% CI were calculated using the Clopper–Pearson method. PFS and OS with 95% CI were assessed using the Kaplan–Meier method. In post hoc exploratory analyses, PFS for each cohort was analyzed by tissue somatic mutation. The association between the HER2 IHC scores and TNBC subtypes was examined using the chi-square test and Fisher’s exact test. Two-tailed Student’s *t*-test, Wilcoxon’s test and Kruskal‒Wallis test were utilized to compare continuous variables where appropriate. All tests were two-sided, and *P* < 0.05 was regarded as statistically significant unless otherwise stated.

SPSS (version 20) and R (version 4.1.1) were used for statistical analysis. The full statistical analysis plan is available in the protocol.

## Supplementary information

Data S1

Supplementary Figure 1

Supplementary Figure 2

Supplementary Figure 3

Supplementary Figure 4

Supplementary Figure 5

Supplementary Figure 6

Supplementary Figure 7

Supplementary Table 1

Supplementary Table 2

Supplementary Table 3

Supplementary Table 4

Supplementary Table 5

Supplementary Table 6

Supplementary Table 7

## References

[1] Waks AG, Winer EP (2019). Breast cancer treatment: a review. JAMA.

[2] Global, regional, and national age-sex-specific mortality and life expectancy, 1950-2017: a systematic analysis for the Global Burden of Disease Study 2017. *Lancet* **392**, 1684–1735 (2018). 10.1016/S0140-6736(18)31891-9 PMC6227504 30496102

[3] Khosravi-Shahi P, Cabezón-Gutiérrez L, Custodio-Cabello S (2018). Metastatic triple negative breast cancer: Optimizing treatment options, new and emerging targeted therapies. Asia Pac. J. Clin. Oncol..

[4] Bardia A (2021). Sacituzumab govitecan in metastatic triple-negative breast cancer. N. Engl. J. Med..

[5] Bianchini G, Balko JM, Mayer IA, Sanders ME, Gianni L (2016). Triple-negative breast cancer: challenges and opportunities of a heterogeneous disease. Nat. Rev. Clin. Oncol..

[6] Schmid P (2018). Atezolizumab and nab-paclitaxel in advanced triple-negative breast cancer. N. Engl. J. Med..

[7] Cortes J (2020). Pembrolizumab plus chemotherapy versus placebo plus chemotherapy for previously untreated locally recurrent inoperable or metastatic triple-negative breast cancer (KEYNOTE-355): a randomised, placebo-controlled, double-blind, phase 3 clinical trial. Lancet.

[8] Liu J (2022). Multicenter phase II trial of Camrelizumab combined with Apatinib and Eribulin in heavily pretreated patients with advanced triple-negative breast cancer. Nat. Commun..

[9] Litton JK (2020). Talazoparib versus chemotherapy in patients with germline BRCA1/2-mutated HER2-negative advanced breast cancer: final overall survival results from the EMBRACA trial. Ann. Oncol..

[10] Robson M (2017). Olaparib for metastatic breast cancer in patients with a germline BRCA mutation. N. Engl. J. Med..

[11] Winer EP (2021). Pembrolizumab versus investigator-choice chemotherapy for metastatic triple-negative breast cancer (KEYNOTE-119): a randomised, open-label, phase 3 trial. Lancet Oncol..

[12] Ma, D. et al. Molecular features and functional implications of germline variants in triple-negative breast cancer. *J. Natl. Cancer Inst.* **113**, 884–892 (2021). 10.1093/jnci/djaa175 33151324

[13] Modi S (2022). Trastuzumab deruxtecan in previously treated HER2-low advanced breast cancer. N. Engl. J. Med..

[14] Turner NC (2020). Circulating tumour DNA analysis to direct therapy in advanced breast cancer (plasmaMATCH): a multicentre, multicohort, phase 2a, platform trial. Lancet Oncol..

[15] Middleton G (2020). The national lung matrix trial of personalized therapy in lung cancer. Nature.

[16] Lee J (2019). Tumor genomic profiling guides patients with metastatic gastric cancer to targeted treatment: The VIKTORY umbrella trial. Cancer Discov..

[17] Rothwell DG (2019). Utility of ctDNA to support patient selection for early phase clinical trials: the TARGET study. Nat. Med..

[18] Jiang YZ (2019). Genomic and transcriptomic landscape of triple-negative breast cancers: subtypes and treatment strategies. Cancer Cell.

[19] Lang GT (2020). Characterization of the genomic landscape and actionable mutations in Chinese breast cancers by clinical sequencing. Nat. Commun..

[20] Lehmann BD (2011). Identification of human triple-negative breast cancer subtypes and preclinical models for selection of targeted therapies. J. Clin. Invest..

[21] Burstein MD (2015). Comprehensive genomic analysis identifies novel subtypes and targets of triple-negative breast cancer. Clin. Cancer Res..

[22] Wu SY, Wang H, Shao ZM, Jiang YZ (2021). Triple-negative breast cancer: new treatment strategies in the era of precision medicine. Sci. China Life Sci..

[23] Zhao S (2020). Molecular subtyping of triple-negative breast cancers by immunohistochemistry: molecular basis and clinical relevance. Oncologist.

[24] Jiang YZ (2021). Molecular subtyping and genomic profiling expand precision medicine in refractory metastatic triple-negative breast cancer: the FUTURE trial. Cell Res..

[25] Berry DA (2011). Adaptive clinical trials in oncology. Nat. Rev. Clin. Oncol..

[26] Xu B (2021). Pyrotinib plus capecitabine versus lapatinib plus capecitabine for the treatment of HER2-positive metastatic breast cancer (PHOEBE): a multicentre, open-label, randomised, controlled, phase 3 trial. Lancet Oncol..

[27] Tarantino P (2022). Antibody-drug conjugates: Smart chemotherapy delivery across tumor histologies. CA Cancer J. Clin..

[28] Voorwerk L (2019). Immune induction strategies in metastatic triple-negative breast cancer to enhance the sensitivity to PD-1 blockade: the TONIC trial. Nat. Med..

[29] Wu SY (2022). Combined angiogenesis and PD-1 inhibition for immunomodulatory TNBC: concept exploration and biomarker analysis in the FUTURE-C-Plus trial. Mol. Cancer.

[30] Chen, L. et al. Famitinib with camrelizumab and nab-paclitaxel for advanced immunomodulatory triple-negative breast cancer (FUTURE-C-PLUS): an open-label, single-arm, phase 2 trial. *Clin. Cancer Res.* **28**, 2807–2817 (2022). 10.1158/1078-0432.CCR-21-4313 PMC9365373 35247906

[31] Hu X (2014). Multicenter phase II study of apatinib, a novel VEGFR inhibitor in heavily pretreated patients with metastatic triple-negative breast cancer. Int. J. Cancer.

[32] Bose R (2013). Activating HER2 mutations in HER2 gene amplification negative breast cancer. Cancer Discov..

[33] Jhaveri K (2022). Abstract GS4-10: Neratinib + fulvestrant + trastuzumab for hormone receptor-positive, HER2-mutant metastatic breast cancer and neratinib + trastuzumab for triple-negative disease: Latest updates from the SUMMIT trial. Cancer Res..

[34] Schmid P (2020). Capivasertib plus paclitaxel versus placebo plus paclitaxel as first-line therapy for metastatic triple-negative breast cancer: The PAKT trial. J. Clin. Oncol..

[35] Kim SB (2017). Ipatasertib plus paclitaxel versus placebo plus paclitaxel as first-line therapy for metastatic triple-negative breast cancer (LOTUS): a multicentre, randomised, double-blind, placebo-controlled, phase 2 trial. Lancet Oncol..

[36] Bartsch R (2021). SABCS 2020: update on triple-negative and metastatic HER2-positive breast cancer. Memo.

[37] Bardia A (2017). Efficacy and safety of anti-Trop-2 antibody drug conjugate sacituzumab govitecan (IMMU-132) in heavily pretreated patients with metastatic triple-negative breast cancer. J. Clin. Oncol..

[38] Bardia A (2021). Biomarker analyses in the phase III ASCENT study of sacituzumab govitecan versus chemotherapy in patients with metastatic triple-negative breast cancer. Ann. Oncol..

[39] Asghar US (2017). Single-cell dynamics determines response to CDK4/6 inhibition in triple-negative breast cancer. Clin. Cancer Res..

[40] Gucalp A (2013). Phase II trial of bicalutamide in patients with androgen receptor-positive, estrogen receptor-negative metastatic Breast Cancer. Clin. Cancer Res..

[41] Bonnefoi H (2016). A phase II trial of abiraterone acetate plus prednisone in patients with triple-negative androgen receptor positive locally advanced or metastatic breast cancer (UCBG 12-1). Ann. Oncol..

[42] Cortes J (2011). Eribulin monotherapy versus treatment of physician’s choice in patients with metastatic breast cancer (EMBRACE): a phase 3 open-label randomised study. Lancet.

[43] Lee JJ, Liu DD (2008). A predictive probability design for phase II cancer clinical trials. Clin. Trials.

[44] Schwartz LH (2016). RECIST 1.1 - standardisation and disease-specific adaptations: perspectives from the RECIST working group. Eur. J. Cancer.

[45] Ding L (2018). Perspective on oncogenic processes at the end of the beginning of cancer genomics. Cell.

[46] Gong, Y. et al. Metabolic-pathway-based subtyping of triple-negative breast cancer reveals potential therapeutic targets. *Cell Metab.* **33**, 51–64.e9 (2021). 10.1016/j.cmet.2020.10.012 33181091

[47] Lewis JS (2005). Intrinsic mechanism of estradiol-induced apoptosis in breast cancer cells resistant to estrogen deprivation. J. Natl Cancer Inst..

[48] Zhu X (2021). Efficacy and mechanism of the combination of PARP and CDK4/6 inhibitors in the treatment of triple-negative breast cancer. J. Exp. Clin. Cancer Res..

[49] Xiao, Y. et al. Comprehensive metabolomics expands precision medicine for triple-negative breast cancer. *Cell Res.* **32**, 477–490 (2022). 10.1038/s41422-022-00614-0 PMC9061756 35105939

[50] Starodub AN (2015). First-in-human trial of a novel anti-Trop-2 antibody-SN-38 conjugate, sacituzumab govitecan, for the treatment of diverse metastatic solid tumors. Clin. Cancer Res..

