# Supplementary material for: Subtyping-based platform guides precision medicine for heavily pretreated metastatic triple-negative breast cancer: The FUTURE phase II umbrella clinical trial
Source: Cell Res. 2023 Mar 27;33(5):389–402. doi: 10.1038/s41422-023-00795-2 (PMC10156707; doi:10.1038/s41422-023-00795-2)
Supplement: Supplementary file 2 — Supplementary Figure 1 [file 41422_2023_795_MOESM2_ESM.pdf]

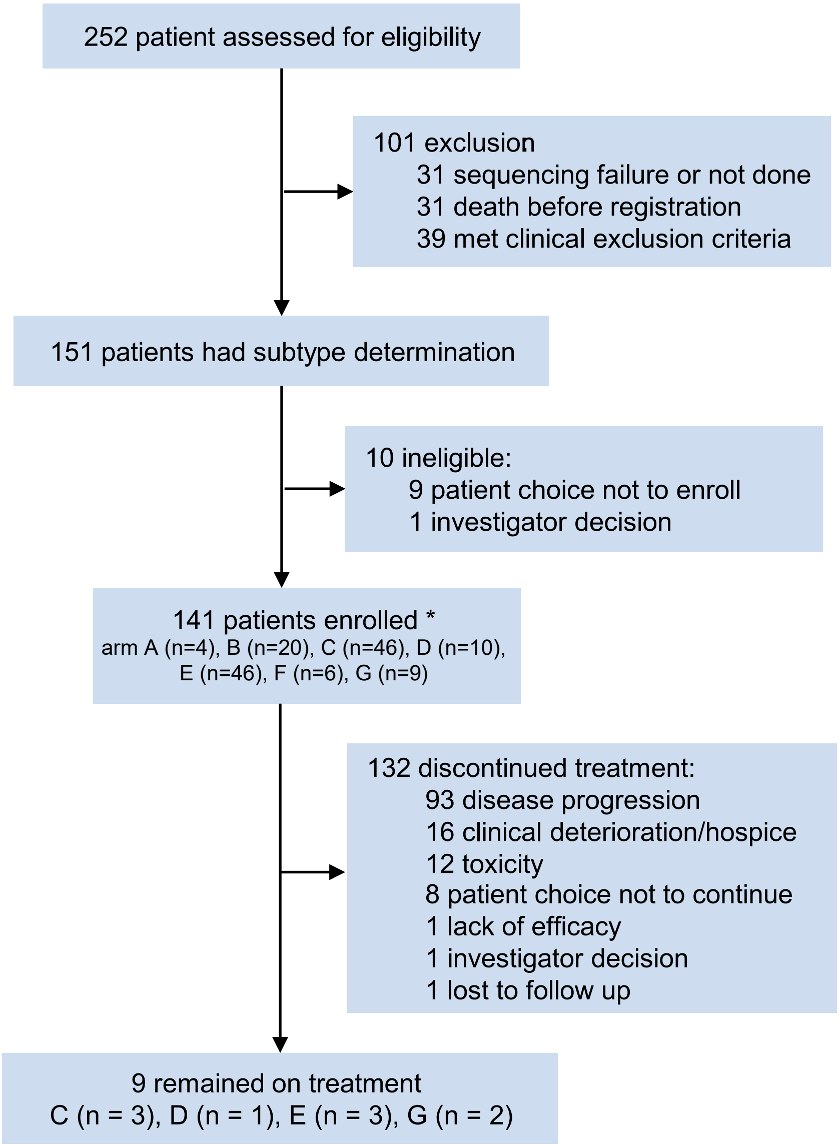

Fig. S1 Trial profile.

*Tumor biopsies were collected to conduct immunohistochemistry staining for triple-negative breast cancer subtyping and next-generation sequencing. Patients were assigned to one of seven parallel treatment arms based on their subtype and sequencing results. n, number.
