# Supplementary material for: Subtyping-based platform guides precision medicine for heavily pretreated metastatic triple-negative breast cancer: The FUTURE phase II umbrella clinical trial
Source: Cell Res. 2023 Mar 27;33(5):389–402. doi: 10.1038/s41422-023-00795-2 (PMC10156707; doi:10.1038/s41422-023-00795-2)
Supplement: Supplementary file 3 — Supplementary Figure 2 [file 41422_2023_795_MOESM3_ESM.pdf]

a

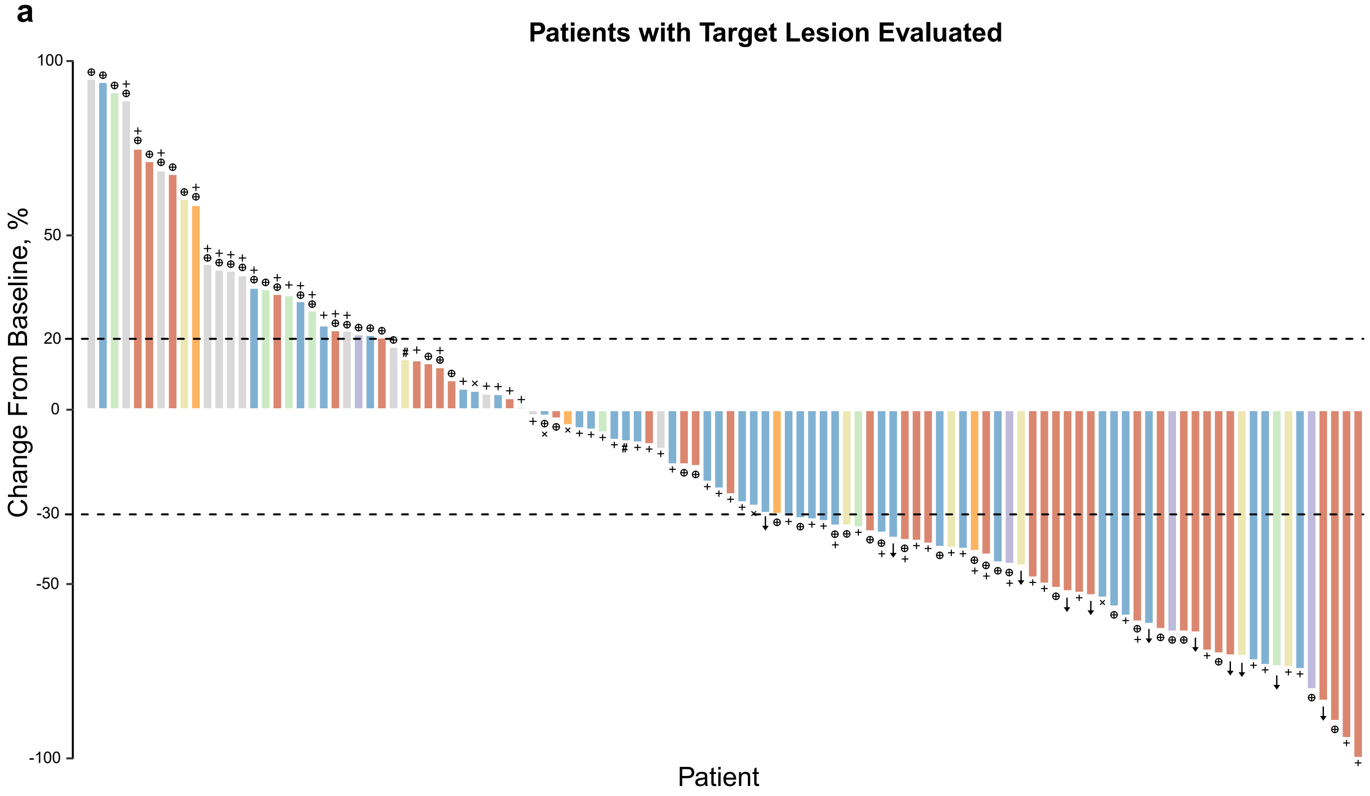

b

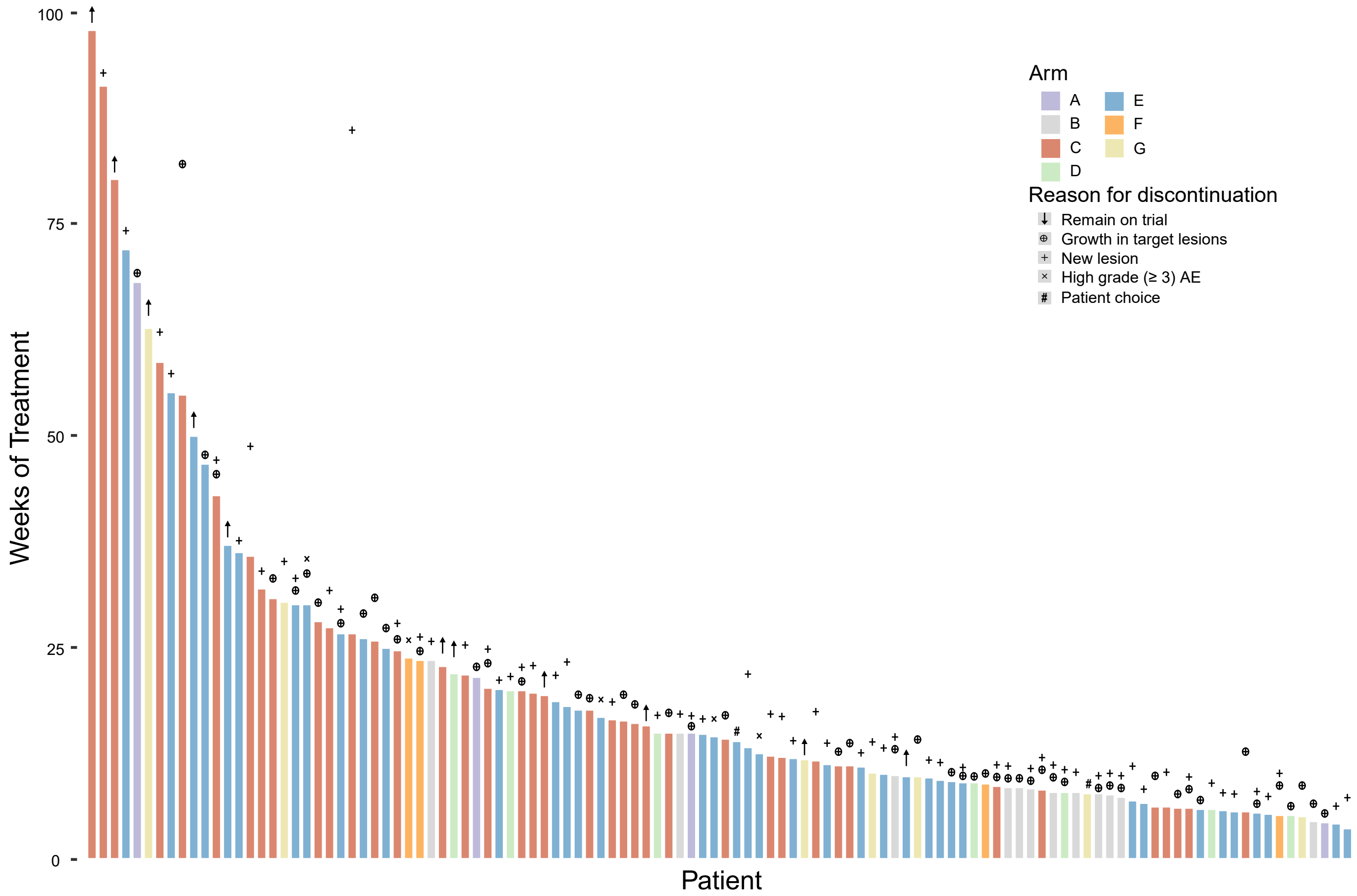

Fig. S2 Summary of response and durability for the whole cohort in the FUTURE trial.
a Waterfall plot that illustrates the best percentage change in the target lesions for patients who had at least one response assessment in the FUTURE trial. Of the 112 patients with at least one postbaseline assessment, two patients with disease progression due to the appearance of new lesions (lack of postbaseline assessment of target lesions) are not shown in the plot. The dashed lines at +20% and -30% indicate thresholds for progressive disease and partial response, respectively, according to RECIST v1.1.
b Duration of treatment for all evaluable patients (n=112).
