# Supplementary material for: Subtyping-based platform guides precision medicine for heavily pretreated metastatic triple-negative breast cancer: The FUTURE phase II umbrella clinical trial
Source: Cell Res. 2023 Mar 27;33(5):389–402. doi: 10.1038/s41422-023-00795-2 (PMC10156707; doi:10.1038/s41422-023-00795-2)
Supplement: Supplementary file 4 — Supplementary Figure 3 [file 41422_2023_795_MOESM4_ESM.pdf]

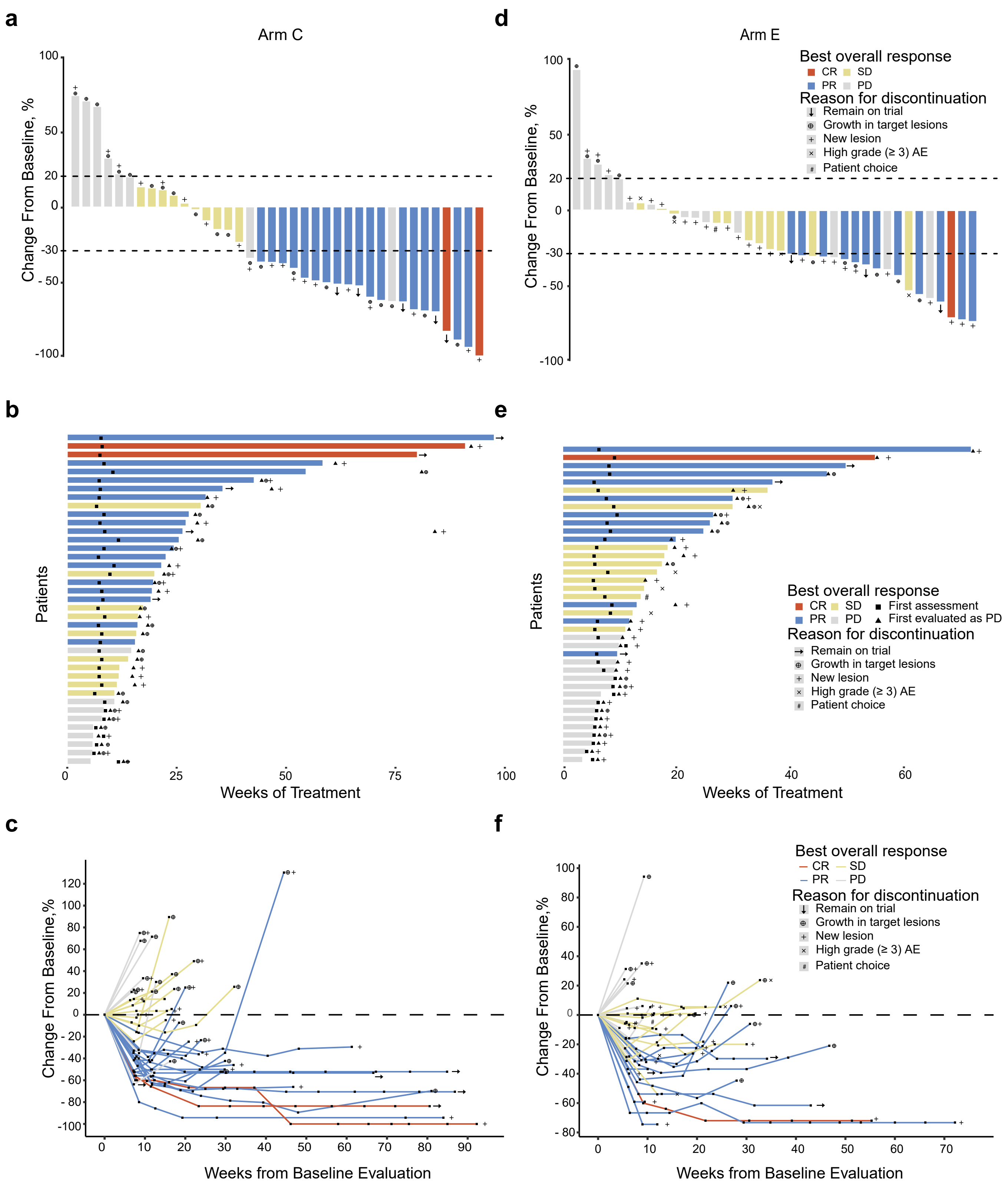

Fig. S3 Summary of response and durability of arm C and E.

a, d Best percent change in the sum of target lesions diameters (longest diameter for non-nodular lesions and short axis for nodal lesions) from baseline in arm C (**a**) and arm E (**d**). Thirty-eight patients in arm C and 38 patients in arm E with postbaseline tumor assessments of target lesions are represented in the figure, while 8 patients in arm C and 8 patients in arm E without postbaseline tumor assessments of target lesions are not shown. The dashed lines at +20% and -30% indicate thresholds for progressive disease and partial response, respectively, according to RECIST v1.1.

b, e Durability of treatment in arm C (**b**) and arm E (**e**).

c, f The longitudinal change in the sum of longest target lesions diameters from baseline in arm C (**c**) and arm E (**f**) with at least one response assessment.

Abbreviations: CR, complete response; PR, partial response; SD, stable disease; PD, progressive disease; AE, adverse event.
