# Supplementary material for: Subtyping-based platform guides precision medicine for heavily pretreated metastatic triple-negative breast cancer: The FUTURE phase II umbrella clinical trial
Source: Cell Res. 2023 Mar 27;33(5):389–402. doi: 10.1038/s41422-023-00795-2 (PMC10156707; doi:10.1038/s41422-023-00795-2)
Supplement: Supplementary file 5 — Supplementary Figure 4 [file 41422_2023_795_MOESM5_ESM.pdf]

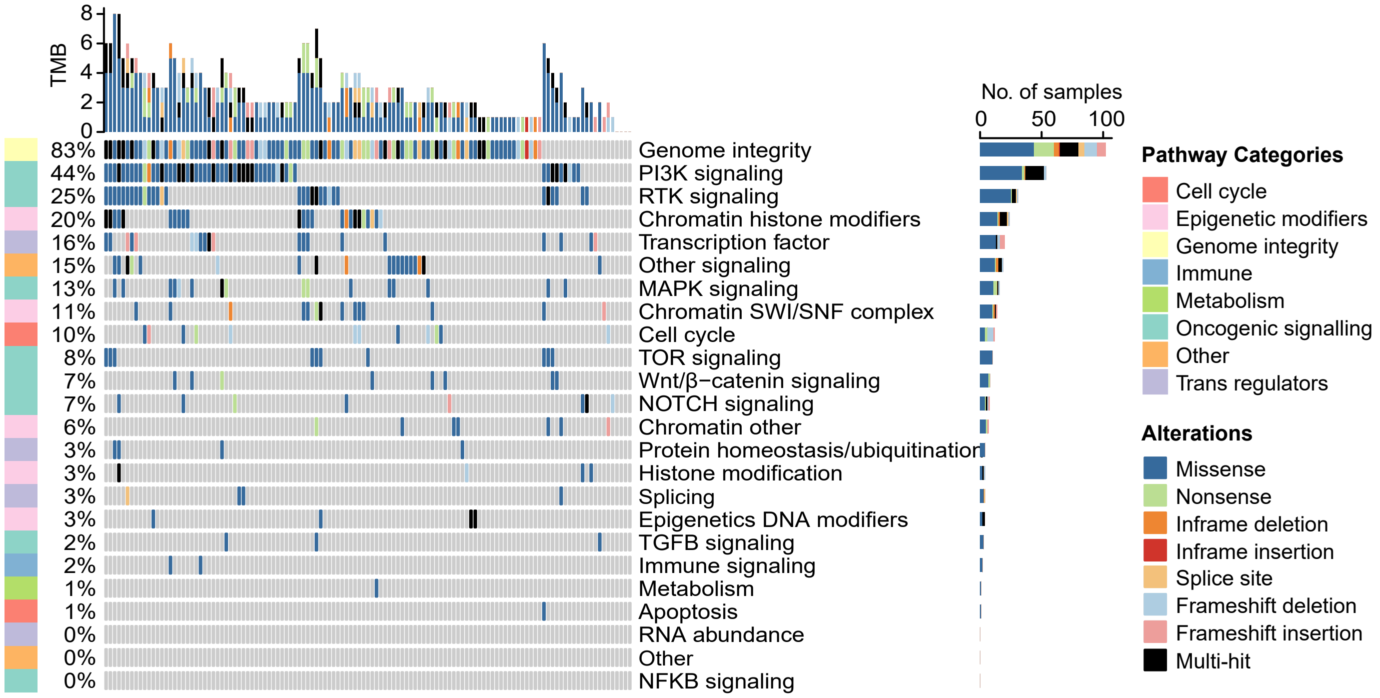

Fig. S4 Characteristics of mutations in oncogenic signaling pathways. The mutation counts in each sample and each pathway are provided above and on the right side, respectively.
