# Supplementary material for: Subtyping-based platform guides precision medicine for heavily pretreated metastatic triple-negative breast cancer: The FUTURE phase II umbrella clinical trial
Source: Cell Res. 2023 Mar 27;33(5):389–402. doi: 10.1038/s41422-023-00795-2 (PMC10156707; doi:10.1038/s41422-023-00795-2)
Supplement: Supplementary file 6 — Supplementary Figure 5 [file 41422_2023_795_MOESM6_ESM.pdf]

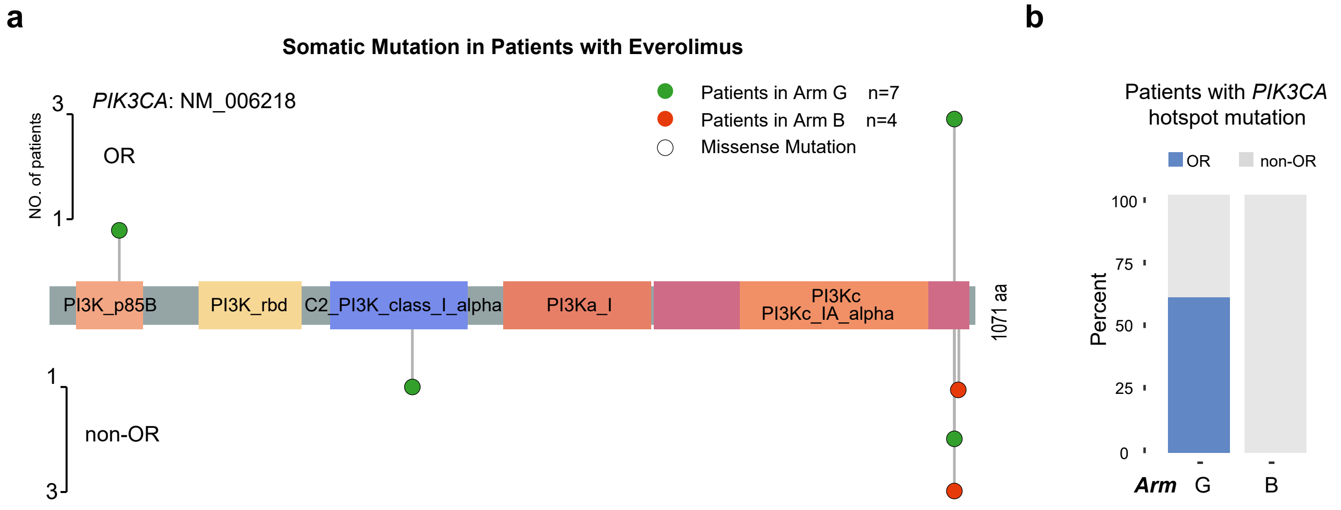

Fig. S5 Somatic PIK3CA mutation site in patients with everolimus in arm B and G.

a Somatic *PIK3CA* mutation sites with different responses of everolimus.

b Objective response rate in patients with *PIK3CA* hotspot mutation H1047.

Abbreviations: OR, objective response.
