# Supplementary material for: Subtyping-based platform guides precision medicine for heavily pretreated metastatic triple-negative breast cancer: The FUTURE phase II umbrella clinical trial
Source: Cell Res. 2023 Mar 27;33(5):389–402. doi: 10.1038/s41422-023-00795-2 (PMC10156707; doi:10.1038/s41422-023-00795-2)
Supplement: Supplementary file 7 — Supplementary Figure 6 [file 41422_2023_795_MOESM7_ESM.pdf]

a

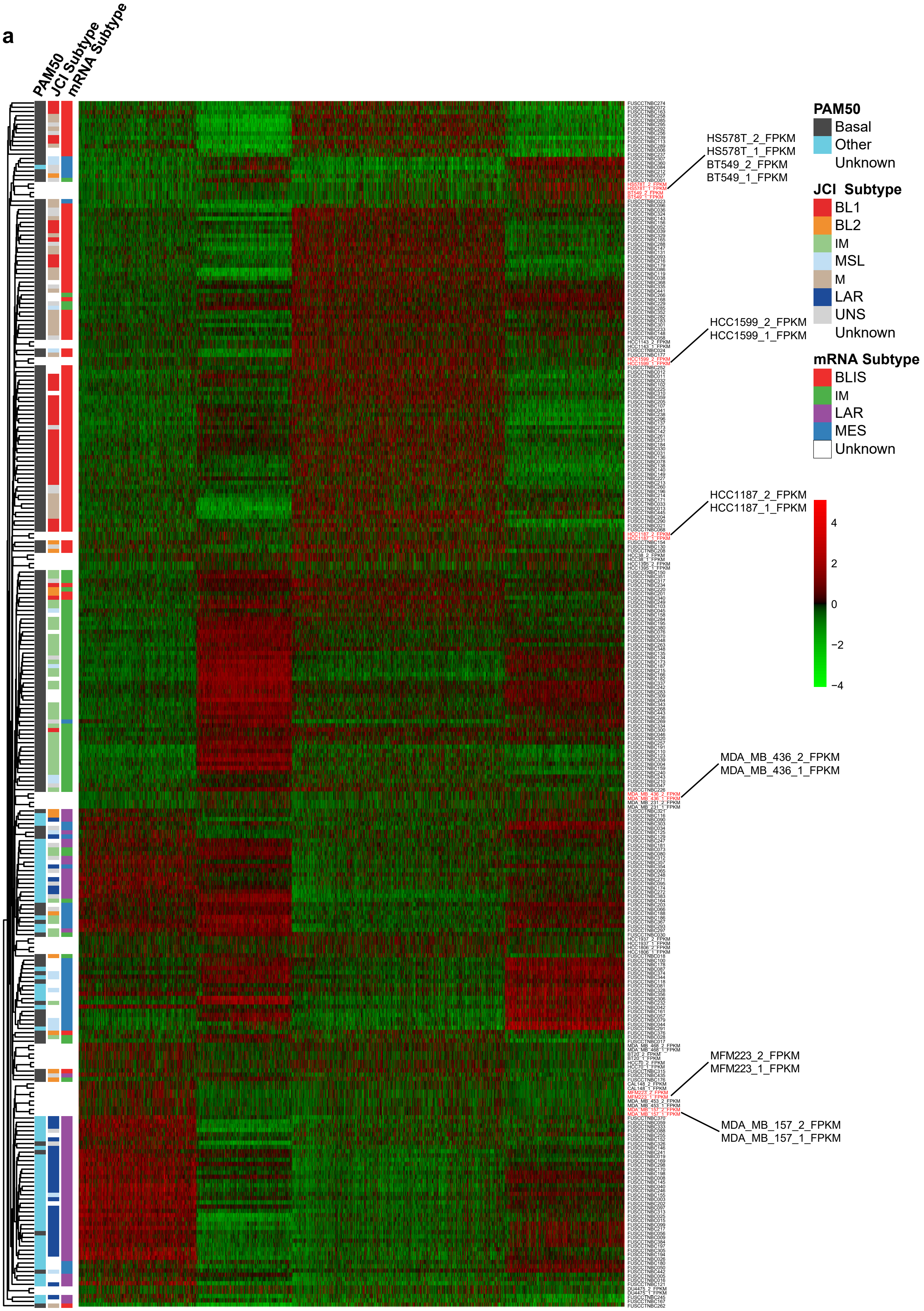
PAM50
JCI Subtype
mRNA Subtype
HS578T_2_FPKM
HS578T_1_FPKM
BT549_2_FPKM
BT549_1_FPKM
HCC1599_2_FPKM
HCC1599_1_FPKM
HCC1187_2_FPKM
HCC1187_1_FPKM
MDA_MB_436_2_FPKM
MDA_MB_436_1_FPKM
MFM223_2_FPKM
MFM223_1_FPKM
MDA_MB_157_2_FPKM
MDA_MB_157_1_FPKM
PAM50
Basal
Other
Unknown
JCI Subtype
BL1
BL2
IM
MSL
M
LAR
UNS
Unknown
mRNA Subtype
BLIS
IM
LAR
MES
Unknown
4
2
0
−2
−4

b

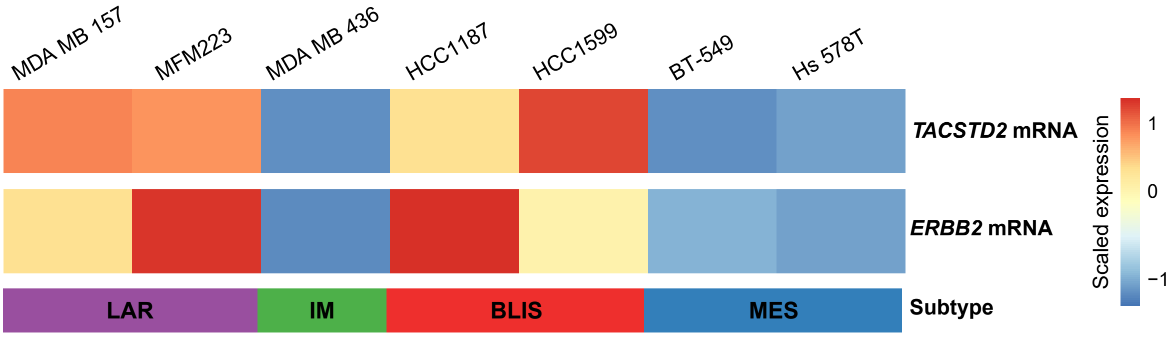

Fig. S6 Characteristics of triple-negative breast cancer cell lines.
a Hierarchical clustering to determine the subtype of TNBC cell lines based on the similarity of expression profiles between patients and cell lines.
b mRNA expression of *ERBB2* and *TACSTD2* in different TNBC cell lines.
Abbreviations: BL, basal-like; IM, immunomodulatory; MSL, mesenchymal stem-like; M, mesenchymal; UNS, unstable; LAR, luminal androgen receptor; BLIS, basal-like immune-suppressed; MES, mesenchymal-like; TNBC, triple-negative breast cancer.
