# Supplementary material for: Subtyping-based platform guides precision medicine for heavily pretreated metastatic triple-negative breast cancer: The FUTURE phase II umbrella clinical trial
Source: Cell Res. 2023 Mar 27;33(5):389–402. doi: 10.1038/s41422-023-00795-2 (PMC10156707; doi:10.1038/s41422-023-00795-2)
Supplement: Supplementary file 8 — Supplementary Figure 7 [file 41422_2023_795_MOESM8_ESM.pdf]

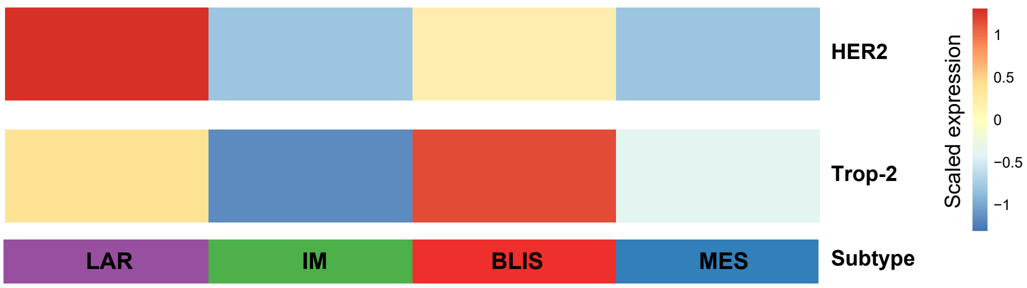

Fig. S7 The expression of HER2 and Trop-2 in patient-derived organoids of triple-negative breast cancer by immunohistochemistry.

Abbreviations: LAR, luminal androgen receptor; IM, immunomodulatory; BLIS, basal-like immune-suppressed; MES, mesenchymal-like.
