# Supplementary material for: Subtyping-based platform guides precision medicine for heavily pretreated metastatic triple-negative breast cancer: The FUTURE phase II umbrella clinical trial
Source: Cell Res. 2023 Mar 27;33(5):389–402. doi: 10.1038/s41422-023-00795-2 (PMC10156707; doi:10.1038/s41422-023-00795-2)
Supplement: Supplementary file 9 — Supplementary Table 1 [file 41422_2023_795_MOESM9_ESM.pdf]

Table S1. The presence of germline BRCA1/2 mutations in the FUTURE trial

	BRCA1	*BRCA2*
	mutant	mutant
A (N = 4)	0	0
B (N = 20)	0	1
C (N = 46)	1	0
D (N = 10)	9	1
E (N = 46)	0	0
F (N = 6)	0	0
G (N = 9)	0	0
Total (N = 141)	10	2
