# Supplementary material for: Subtyping-based platform guides precision medicine for heavily pretreated metastatic triple-negative breast cancer: The FUTURE phase II umbrella clinical trial
Source: Cell Res. 2023 Mar 27;33(5):389–402. doi: 10.1038/s41422-023-00795-2 (PMC10156707; doi:10.1038/s41422-023-00795-2)
Supplement: Supplementary file 10 — Supplementary Table 2 [file 41422_2023_795_MOESM10_ESM.pdf]

Table S2. The expression of PD-L1 in the FUTURE trial

	PD-L1	
	CPS <10	CPS ≥10
A (N = 3)	3	0
B (N = 12)	8	4
C (N = 15)	2	13
D (N = 9)	8	1
E (N = 28)	25	3
F (N = 5)	5	0
G (N = 6)	6	0
Total (N = 78*)	57	21

* A total of 78 patients in the FUTURE trial had available FFPE sections and were successfully stained for PD-L1 (clone 22C3) by immunohistochemistry.

Abbreviations: CPS, combined positive score.
