# Supplementary material for: Subtyping-based platform guides precision medicine for heavily pretreated metastatic triple-negative breast cancer: The FUTURE phase II umbrella clinical trial
Source: Cell Res. 2023 Mar 27;33(5):389–402. doi: 10.1038/s41422-023-00795-2 (PMC10156707; doi:10.1038/s41422-023-00795-2)
Supplement: Supplementary file 11 — Supplementary Table 3 [file 41422_2023_795_MOESM11_ESM.pdf]

Table S3. Summary of 29 Patients without one on treatment assessment in ITT population of the FUTURE trial

	ITT (N=141)	
	N	%
**With at least one on treatment assessment**	112	79.4
**Without one on treatment assessment**	29	20.6
**Adverse event**	3	2.1
**Patient choice**	9	6.4
**Clinical deterioration**	17	12.1
