# Supplementary material for: Subtyping-based platform guides precision medicine for heavily pretreated metastatic triple-negative breast cancer: The FUTURE phase II umbrella clinical trial
Source: Cell Res. 2023 Mar 27;33(5):389–402. doi: 10.1038/s41422-023-00795-2 (PMC10156707; doi:10.1038/s41422-023-00795-2)
Supplement: Supplementary file 12 — Supplementary Table 4 [file 41422_2023_795_MOESM12_ESM.pdf]

Table S4. Summary of survival in the FUTURE trial

	ITT (N = 141)	A (n = 4)	B (n = 20)	C (n = 46)	D (n = 10)	E (n = 46)	F (n = 6)	G (n = 9)
Median PFS (95% CI), month	3.4 (2.7-4.2)	3.4 (0-7.3)	1.9 (1.7-2.1)	4.6 (3.4-5.9)	2.0 (1.7-2.3)	3.4 (1.7-5.0)	1.2 (0-2.5)	3.0 (2.4-3.6)
Event, No. of patients (%)	111 (78.7)	4 (100.0)	14 (70.0)	36 (78.3)	8 (80.0)	39 (84.8)	5 (83.3)	5 (55.6)
Censoring, No. of patients (%)	30 (21.3)	0	6 (30.0)	10 (21.7)	2 (20.0)	7 (15.2)	1 (16.7)	4 (44.4)
Median OS (95% CI), month	10.7 (9.1-12.3)	16.7 (0-35.3)	6.1 (2.8-9.4)	16.1 (11.7-20.5)	6.2 (1.9-10.5)	10.1 (3.8-16.3)	2.7 (0-17.0)	4.5 (2.4-6.6)
Event, No. of patients (%)	90 (63.8)	3 (75.0)	17 (85.0)	23 (50.0)	8 (80.0)	28 (60.9)	5 (83.3)	6 (66.7)
Censoring, No. of patients (%)	51 (36.2)	1 (25.0)	3 (15.0)	23 (50.0)	2 (20.0)	18 (39.1)	1 (16.7)	3 (33.3)

Abbreviations: ITT, intention-to-treat; CI, confidence interval; PFS, progression free survival; OS, overall survival.
