# Supplementary material for: Subtyping-based platform guides precision medicine for heavily pretreated metastatic triple-negative breast cancer: The FUTURE phase II umbrella clinical trial
Source: Cell Res. 2023 Mar 27;33(5):389–402. doi: 10.1038/s41422-023-00795-2 (PMC10156707; doi:10.1038/s41422-023-00795-2)
Supplement: Supplementary file 13 — Supplementary Table 5 [file 41422_2023_795_MOESM13_ESM.pdf]

Table S5. Treatment-Related (including definite, possible, and probable) Adverse Events of Any Grade and Grades 3 to 4 Occurring in Each Arm

Adverse event	A (n = 4)				B (n = 20)				C (n = 46)				D (n = 10)				E (n = 46)				F (n = 6)				G (n = 9)				Summary (n = 141)					
	Any Grade		Grade≥3		Any Grade		Grade≥3		Any Grade		Grade≥3		Any Grade		Grade≥3		Any Grade		Grade≥3		Any Grade		Grade≥3		Any Grade		Grade≥3		Any Grade		Grade 1-2		Grade≥3	
	n	%	n	%	n	%	n	%	n	%	n	%	n	%	n	%	n	%	n	%	n	%	n	%	n	%	n	%	n	%	n	%	n	%
Nausea	1	25.0	0	0.0	2	10.0	0	0.0	6	13.0	0	0.0	5	50.0	0	0.0	18	39.1	0	0.0	1	16.7	0	0.0	0	0.0	0	0.0	33	23.4	33	23.4	0	0.0
Anorexia	2	50.0	0	0.0	0	0.0	0	0.0	2	4.3	1	2.2	1	25.0	0	0.0	12	26.1	2	4.3	4	66.7	0	0.0	0	0.0	0	0.0	21	14.9	18	12.8	3	2.1
Vomiting	1	25.0	0	0.0	2	10.0	0	0.0	4	8.7	0	0.0	3	30.0	0	0.0	10	21.7	0	0.0	0	0.0	0	0.0	0	0.0	0	0.0	20	14.2	20	14.2	0	0.0
Diarrhea	3	75.0	0	0.0	2	10.0	1	5.0	3	6.5	0	0.0	0	0.0	0	0.0	6	13.0	0	0.0	0	0.0	0	0.0	0	0.0	0	0.0	14	9.9	13	9.2	1	0.7
Constipation	0	0.0	0	0.0	0	0.0	0	0.0	1	2.1	0	0.0	1	10.0	0	0.0	1	2.2	0	0.0	0	0.0	0	0.0	0	0.0	0	0.0	3	2.1	3	2.1	0	0.0
Abdominal pain	0	0.0	0	0.0	1	5.0	0	0.0	0	0.0	0	0.0	0	0.0	0	0.0	2	4.3	0	0.0	0	0.0	0	0.0	0	0.0	0	0.0	3	2.1	3	2.1	0	0.0
Dyspnea	0	0.0	0	0.0	1	7.0	1	7.1	1	2.1	0	0.0	0	0.0	0	0.0	3	6.5	1	2.2	1	16.7	0	0.0	0	0.0	0	0.0	6	4.3	5	3.5	1	0.7
Cough	0	0.0	0	0.0	0	0.0	0	0.0	2	4.3	0	0.0	0	0.0	0	0.0	5	10.9	0	0.0	2	33.3	0	0.0	0	0.0	0	0.0	9	6.4	9	6.4	0	0.0
Fatigue	1	25.0	0	0.0	3	15.0	0	0.0	12	26.1	0	0.0	1	10.0	0	0.0	19	41.3	0	0.0	2	33.3	0	0.0	2	22.2	0	0.0	40	28.4	40	28.4	0	0.0
Pyrexia	0	0.0	0	0.0	0	0.0	0	0.0	5	10.9	1	2.2	0	0.0	0	0.0	1	2.2	0	0.0	1	16.7	0	0.0	1	11.1	1	11.1	8	5.7	6	4.3	2	1.4
Weight loss	2	50.0	0	0.0	0	0.0	0	0.0	2	4.3	0	0.0	0	0.0	0	0.0	8	17.4	0	0.0	0	0.0	0	0.0	1	11.1	0	0.0	13	9.2	13	9.2	0	0.0
Weight gain	0	0.0	0	0.0	0	0.0	0	0.0	1	2.2	0	0.0	0	0.0	0	0.0	0	0.0	0	0.0	0	0.0	0	0.0	0	0.0	0	0.0	1	0.7	1	0.7	0	0.0
Rash	0	0.0	0	0.0	0	0.0	0	0.0	6	13.0	1*	2.2	0	0.0	0	0.0	4	8.7	0	0.0	0	0.0	0	0.0	0	0.0	0	0.0	10	7.1	9	6.4	1	0.7
Arthralgia	0	0.0	0	0.0	1	5.0	0	0.0	2	4.3	0	0.0	1	10.0	0	0.0	2	4.3	0	0.0	0	0.0	0	0.0	0	0.0	0	0.0	6	4.3	6	4.3	0	0.0
Myalgia	0	0.0	0	0.0	0	0.0	0	0.0	3	6.5	0	0.0	1	10.0	0	0.0	0	0.0	0	0.0	0	0.0	0	0.0	0	0.0	0	0.0	4	2.8	4	2.8	0	0.0
Back pain	0	0.0	0	0.0	1	5.0	0	0.0	0	0.0	0	0.0	0	0.0	0	0.0	1	2.2	0	0.0	1	16.7	1	16.7	0	0.0	0	0.0	3	2.1	2	1.4	1	0.7
Pruritus	0	0.0	0	0.0	1	5.0	0	0.0	1	2.2	0	0.0	0	0.0	0	0.0	1	2.2	0	0.0	0	0.0	0	0.0	0	0.0	0	0.0	3	2.1	3	2.1	0	0.0
Mucositis	2	50.0	0	0.0	2	10.0	0	0.0	3	6.5	0	0.0	1	10.0	0	0.0	6	13.0	0	0.0	3	50.0	0	0.0	3	33.3	0	0.0	20	14.2	20	14.2	0	0.0
Hoarseness	0	0.0	0	0.0	0	0.0	0	0.0	1	2.2	0	0.0	0	0.0	0	0.0	1	2.2	0	0.0	0	0.0	0	0.0	0	0.0	0	0.0	2	1.4	2	1.4	0	0.0
PPE	1	25.0	0	0.0	0	0.0	0	0.0	2	4.3	0	0.0	0	0.0	0	0.0	17	37.0	4	8.7	1	16.7	0	0.0	0	0.0	0	0.0	20	14.2	17	12.1	3	2.1
Peripheral neuropathy	1	25.0	0	0.0	3	15.0	0	0.0	20	43.5	0	0.0	0	0.0	0	0.0	9	19.6	0	0.0	0	0.0	0	0.0	1	11.0	0	0.0	34	24.1	34	24.1	0	0.0
Pneumonia	0	0.0	0	0.0	1	5.0	0	0.0	2	4.3	0	0.0	0	0.0	0	0.0	1	2.2	0	0.0	0	0.0	0	0.0	4	44.4	2	22.2	8	5.7	6	4.3	2	1.4
Hypertension	0	0.0	0	0.0	0	0.0	0	0.0	2	4.3	0	0.0	1	10.0	0	0.0	22	47.8	6	13.0	3	50.0	1	16.7	1	0.0	0	0.0	29	20.6	22	15.6	7	5.0
Anemia	1	25.0	0	0.0	8	40.0	4	20.0	29	63.0	5	10.9	5	50.0	1	10.0	25	54.3	3	6.5	4	66.7	1	16.7	5	55.6	3	33.3	78	55.3	61	43.3	17	12.1
Leukopenia	1	25.0	0	0.0	6	30.0	0	0.0	34	73.9	12	26.1	4	40.0	1	10.0	25	54.3	6	13.0	4	66.7	1	16.7	8	88.9	2	22.2	82	58.2	60	42.6	22	15.6
Neutropenia	1	25.0	0	0.0	3	15.0	1	5.0	23	50.0	11	23.9	3	30.0	2	20.0	15	32.6	4	8.7	4	66.7	2	33.3	2	22.2	1	11.1	51	36.2	30	21.3	21	14.9
Thrombocytopenia	1	25.0	0	0.0	6	30.0	3	15.0	12	26.1	3	6.5	5	50.0	4	40.0	16	34.8	2	4.3	3	50.0	1	16.7	5	55.6	1	0.0	48	34.0	34	24.1	14	9.9
Elevated ALT/AST	1	25.0	0	0.0	5	25.0	0	0.0	10	21.7	2	4.3	0	0.0	0	0.0	13	28.3	2	4.3	3	50.0	0	0.0	3	33.3	0	0.0	35	24.8	32	22.7	3	2.1
Proteinuria	0	0.0	0	0.0	0	0.0	0	0.0	1	2.2	0	0.0	0	0.0	0	0.0	16	34.8	4	8.7	5	83.3	1	16.7	0	0.0	0	0.0	21	14.9	17	12.1	4	2.8
Urinary occult blood	0	0.0	0	0.0	0	0.0	0	0.0	0	0.0	0	0.0	1	10.0	1	10.0	0	0.0	0	0.0	1	16.7	0	0.0	0	0.0	0	0.0	2	1.4	1	0.7	1	0.7
RCCEP	0	0.0	0	0.0	0	0.0	0	0.0	28	60.9	1	2.2	0	0.0	0	0.0	0	0.0	0	0.0	0	0.0	0	0.0	0	0.0	0	0.0	28	19.9	27	19.1	1	0.7
Allergic purpura	0	0.0	0	0.0	1	5.0	0	0.0	0	0.0	0	0.0	0	0.0	0	0.0	0	0.0	0	0.0	0	0.0	0	0.0	0	0.0	0	0.0	1	0.7	1	0.7	0	0.0
Hypoalbuminemia	0	0.0	0	0.0	0	0.0	0	0.0	1	2.2	0	0.0	0	0.0	0	0.0	1	2.2	0	0.0	0	0.0	0	0.0	0	0.0	0	0.0	2	1.4	2	1.4	0	0.0
Sepsis	0	0.0	0	0.0	1	5.0	0	0.0	0	0.0	0	0.0	0	0.0	0	0.0	0	0.0	0	0.0	0	0.0	0	0.0	0	0.0	0	0.0	1	0.7	0	0.0	1	0.7
Hypothyroidism	0	0.0	0	0.0	0	0.0	0	0.0	4	8.7	1	2.2	0	0.0	0	0.0	2	4.3	0	0.0	0	0.0	0	0.0	0	0.0	0	0.0	6	4.3	5	3.5	1	0.7
Infusion related reaction	0	0.0	0	0.0	0	0.0	0	0.0	4	8.7	0	0.0	0	0.0	0	0.0	0	0.0	0	0.0	0	0.0	0	0.0	0	0.0	0	0.0	4	2.8	4	2.8	0	0.0
Blood bilirubin increased	0	0.0	0	0.0	0	0.0	0	0.0	2	4.3	0	0.0	0	0.0	0	0.0	3	6.5	0	0.0	0	0.0	0	0.0	0	0.0	0	0.0	4	2.8	4	2.8	0	0.0
Creatinine increased	0	0.0	0	0.0	0	0.0	0	0.0	1	2.2	0	0.0	1	10.0	0	0.0	1	2.2	0	0.0	0	0.0	0	0.0	0	0.0	0	0.0	3	2.1	3	2.1	0	0.0
Hyperglycemia	0	0.0	0	0.0	0	0.0	0	0.0	2	4.3	1	2.2	0	0.0	0	0.0	1	2.2	0	0.0	0	0.0	0	0.0	0	0.0	0	0.0	3	2.1	2	1.4	1	0.7
Hypokalemia	1	25.0	1	25.0	0	0.0	0	0.0	0	0.0	0	0.0	0	0.0	0	0.0	0	0.0	0	0.0	0	0.0	0	0.0	0	0.0	0	0.0	1	0.7	0	0.0	1	0.7
stroke	0	0.0	0	0.0	0	0.0	0	0.0	0	0.0	0	0.0	0	0.0	0	0.0	1	2.2	1	2.2	0	0.0	0	0.0	0	0.0	0	0.0	1	0.7	0	0.0	1	0.7
Edema limbs	0	0.0	0	0.0	0	0.0	0	0.0	7	15.2	0	0.0	0	0.0	0	0.0	2	4.3	0	0.0	0	0.0	0	0.0	0	0.0	0	0.0	9	6.4	9	6.4	0	0.0
Skin Ulceration	0	0.0	0	0.0	0	0.0	0	0.0	0	0.0	0	0.0	0	0.0	0	0.0	3	6.5	0	0.0	0	0.0	0	0.0	0	0.0	0	0.0	3	2.1	0	0.0	3	2.1

Thrombosis	0	0.0	0	0.0	0	0.0	0	0.0	2	4.3	0	0.0	0	0.0	0	0.0	0	0.0	0	0.0	0	0.0	0	0.0	0	0.0	0	0.0	2	1.4	2	1.4	0	0.0

Abbreviations: ALT, alanine aminotransferase; AST, aspartate transaminase; PPE, Palmar-plantar erythrodysesthesia; RCCEP, reactive cutaneous capillary endothelial proliferation.
