# Supplementary material for: Subtyping-based platform guides precision medicine for heavily pretreated metastatic triple-negative breast cancer: The FUTURE phase II umbrella clinical trial
Source: Cell Res. 2023 Mar 27;33(5):389–402. doi: 10.1038/s41422-023-00795-2 (PMC10156707; doi:10.1038/s41422-023-00795-2)
Supplement: Supplementary file 14 — Supplementary Table 6 [file 41422_2023_795_MOESM14_ESM.pdf]

Table S6. Subgroup analysis of confirmed ORR in ITT population

Characteristic	OR (N)	Non-OR (N)	ORR, 95% CI (%)
Age, years			
10-50	14	56	20.0 (11.4-31.3)
51-70	28	43	39.4 (28.0-51.7)
Number of metastatic organs			
1-3	35	65	35.0 (25.7-45.2)
>3	7	34	17.1 (7.2-32.1)
ECOG			
0-1	40	85	32.0 (23.9-40.9)
2	2	14	12.5 (1.6-38.3)
DFI (months)			
<6	11	42	20.8 (10.8-34.1)
≥6	23	41	35.9 (24.3-48.9)
NA (Stage IV)	8	16	33.3 (15.6-55.3)
First-line PFS (months)			
<3	13	35	27.1 (15.3-41.8)
3-6	11	31	26.2 (13.9-42.0)
>6	15	20	42.9 (26.3-60.6)
unknown	3	13	18.8 (4.0-45.6)
Previous lines of treatment			
<3	15	41	26.8 (15.8-40.3)
≥3	27	58	31.8 (22.1-42.8)

Abbreviations: ITT, intention-to-treat; ORR, objective response rate; OR, objective response; CI, confidence interval; N, number; ECOG, Eastern Cooperative Oncology Group; DFI, disease free interval; NA, not available; PFS, progression free survival.
