# Supplementary material for: Subtyping-based platform guides precision medicine for heavily pretreated metastatic triple-negative breast cancer: The FUTURE phase II umbrella clinical trial
Source: Cell Res. 2023 Mar 27;33(5):389–402. doi: 10.1038/s41422-023-00795-2 (PMC10156707; doi:10.1038/s41422-023-00795-2)
Supplement: Supplementary file 15 — Supplementary Table 7 [file 41422_2023_795_MOESM15_ESM.pdf]

Table S7. Sequencing gene content of the prospective cohort.

ABCB1	*BTK*	*CRIP1*	*FER*	*HRAS*	*KMT2B*	*MEN1*	*OR2L2*	*PREX2*	*RPGR*	*TEP1*
ABL1	*BUB1B*	*CRK*	*FES*	*HSP90AA1*	*KMT2C*	*MERTK*	*OR6A2*	*PRKAA1*	*RPS6KA1*	*TERT*
ADA	*CACNA1D*	*CSF1R*	*FGFR1*	*HSP90AB1*	*KMT2D*	*MET*	*PAK1*	*PRKACA*	*RPS6KA3*	*TET2*
AFF2	*CAMK2G*	*CSF3R*	*FGFR2*	*HSP90B1*	*KRAS*	*MICA*	*PAK2*	*PRKACB*	*RPS6KA5*	*TGFBR1*
AKAP3	*CASP8*	*CSNK1E*	*FGFR3*	*HSPA4*	*LCK*	*MIEN1*	*PAK3*	*PRKCA*	*RPS6KB1*	*TKTL1*
AKT1	*CAV3*	*CSNK2A1*	*FGFR4*	*IGF1*	*LPL*	*MLH1*	*PALB2*	*PRKCB*	*RPTOR*	*TLR4*
AKT2	*CBFB*	*CTCF*	*FGR*	*IGF1R*	*LYN*	*MLLT4*	*PARP1*	*PRKCD*	*RRM1*	*TLR9*
AKT3	*CBL*	*CTCFL*	*FH*	*IKBKB*	*MAP1A*	*MMP1*	*PARP2*	*PRKCE*	*RRM2*	*TNFRSF11A*
ALDH1A1	*CBLB*	*CUL4A*	*FLT1*	*IL15RA*	*MAP1B*	*MMP14*	*PBRM1*	*PRKCG*	*RUNX1*	*TNK2*
ALK	*CCL1*	*CUX1*	*FLT3*	*IL2*	*MAP2*	*MMP2*	*PCID2*	*PRKCQ*	*RYR2*	*TNKS2*
ALOX5	*CCL2*	*CYP19A1*	*FLT4*	*IL7R*	*MAP2K1*	*MMP3*	*PDE4B*	*PRKCZ*	*S100B*	*TOP1*
ANKRD11	*CCL20*	*CYP2C8*	*FOXA1*	*INSR*	*MAP2K2*	*MMP9*	*PDE4C*	*PRKDC*	*S1PR2*	*TOP2A*
APC	*CCL7*	*CYP2C9*	*FOXO3*	*INSRR*	*MAP2K4*	*MSH2*	*PDE4D*	*PRKG1*	*SETD2*	*TOP2B*
APP	*CCL8*	*CYP3A4*	*FOXP1*	*IRAK1*	*MAP3K1*	*MST1R*	*PDGFB*	*PRKX*	*SF3B1*	*TOP3A*
ARAF	*CCND1*	*DCAF4L2*	*FOXQ1*	*IRF4*	*MAP3K13*	*MTAP*	*PDGFRA*	*PRMT2*	*SGK1*	*TOP3B*
ARID1A	*CCND2*	*DEK*	*FRK*	*IRS1*	*MAP3K2*	*MTHFR*	*PDGFRB*	*PTEN*	*SGK2*	*TP53*
ARID1B	*CCND3*	*DGKG*	*FSIP1*	*IRS2*	*MAP3K4*	*MTOR*	*PDPK1*	*PTGS2*	*SHC1*	*TPMT*
ARID2	*CCNE1*	*DNMT1*	*FYN*	*ITCH*	*MAP3K5*	*MYB*	*PEG10*	*PTK2*	*SHH*	*TPRX1*
ASXL1	*CD274*	*DNMT3A*	*GAB2*	*ITGB3*	*MAP3K6*	*MYC*	*PFKFB3*	*PTK2B*	*SIK2*	*TRIM47*
ATM	*CDC25A*	*DNMT3B*	*GATA3*	*ITGB4*	*MAP3K7*	*NCOA1*	*PGR*	*PTK6*	*SIRT1*	*TRIM65*
ATN1	*CDC25B*	*DUSP7*	*GHRL*	*ITK*	*MAP4*	*NCOR1*	*PHF6*	*PTPN22*	*SIRT7*	*TRIM6-TRIM34*
ATP2B2	*CDC25C*	*DYRK1A*	*GLIS3*	*JAK1*	*MAP4K3*	*NEK2*	*PHKA2*	*PTPRD*	*SLC19A3*	*TRPS1*
ATR	*CDH1*	*ECT2L*	*GNAS*	*JAK2*	*MAP4K4*	*NF1*	*PI4K2A*	*PTPRN2*	*SLC5A1*	*TSC1*
ATRX	*CDH16*	*EGFR*	*GOLPH3L*	*JAK3*	*MAP4K5*	*NF2*	*PI4KB*	*PVT1*	*SMAD4*	*TSC2*
AURKA	*CDK16*	*EHMT2*	*GPR32*	*JUN*	*MAP7*	*NFIB*	*PIK3C3*	*RAB25*	*SMARCA4*	*TTK*
AURKB	*CDK2*	*EPAS1*	*GPS2*	*KAT6A*	*MAPK1*	*NFKB1*	*PIK3CA*	*RAC1*	*SMO*	*TUSC5*
AURKC	*CDK4*	*EPHA1*	*GRB7*	*KAT8*	*MAPK10*	*NFKB2*	*PIK3CB*	*RAC2*	*SMYD3*	*TYK2*
AXIN1	*CDK6*	*EPHA2*	*GRIN2A*	*KCNB2*	*MAPK13*	*NFKBIA*	*PIK3CD*	*RAD18*	*SOCS2*	*TYRO3*
AXL	*CDKN1B*	*EPHA3*	*GSK3A*	*KCNJ12*	*MAPK14*	*NOS1*	*PIK3CG*	*RAD20*	*SOX9*	*UNC13D*
BAK1	*CDKN2A*	*EPHB1*	*GSK3B*	*KCNJ14*	*MAPK3*	*NOS2*	*PIK3R1*	*RAD51*	*SPEN*	*USH2A*
BAX	*CDKN2B*	*EPHB2*	*HDAC1*	*KCNJ2*	*MAPK4*	*NOS3*	*PIK3R2*	*RAF1*	*SRC*	*USP9X*
BCL2	*CETP*	*EPHB4*	*HDAC2*	*KCNJ3*	*MAPK6*	*NOTCH1*	*PIKFYVE*	*RASAL1*	*SRMS*	*VHL*
BCL2A1	*CHEK1*	*EPSTI1*	*HDAC4*	*KCNJ5*	*MAPK7*	*NOTCH2*	*PIM1*	*RB1*	*SSTR5*	*VPS4B*
BCL2L2	*CHEK2*	*ERBB2*	*HDAC5*	*KCNJ6*	*MAPK8*	*NOTCH3*	*PIWIL1*	*RBM17*	*ST8SIA4*	*WEE1*
BCOR	*CHML*	*ERBB3*	*HDAC6*	*KCNJ9*	*MAPRE2*	*NR1H2*	*PKD1*	*RECQL5*	*STAG2*	*WHSC1L1*
BCR	*CHRNA4*	*ERBB4*	*HDAC7*	*KDM1B*	*MAPRE3*	*NR1H4*	*PLCG1*	*REL*	*STAT1*	*WNK3*
BIRC2	*CHUK*	*ERCC4*	*HDAC9*	*KDM4A*	*MAPT*	*NR2F2*	*PLK2*	*RET*	*STAT5A*	*WNT7A*
BIRC3	*CIC*	*ESR1*	*HIF1A*	*KDM5A*	*MAST2*	*NR3C1*	*PLK3*	*RFC1*	*STAT5B*	*XBP1*
BLK	*CLEC19A*	*EXO1*	*HIST1H1C*	*KDM6A*	*MCL1*	*NRAS*	*PMS2*	*RGMB*	*STK11*	*XDH*
BMP7	*CNOT3*	*FAAH*	*HIST1H2BC*	*KDR*	*MDM2*	*NTRK1*	*PNPLA3*	*RHOA*	*SYK*	*YES1*
BMX	*CNR1*	*FAM47C*	*HIST1H3B*	*KIF11*	*MDM4*	*NTRK2*	*PPAPDC1B*	*RICTOR*	*TBL1XR1*	*ZAP70*
BRAF	*COL1A1*	*FANCD2*	*HK2*	*KIF26B*	*MECOM*	*OGG1*	*PPARA*	*RIPK1*	*TBX3*	*ZBP1*
BRCA1	*COL2A1*	*FASN*	*HK3*	*KIT*	*MECP2*	*OPCML*	*PPARG*	*ROCK1*	*TEC*	*ZFP36L1*
BRCA2	*CREBBP*	*FBXW7*	*HMGCR*	*KLLN*	*MED23*	*OR2G3*	*PRDM1*	*ROCK2*	*TEK*	*ZHX2*
